# Development of a High-Resolution Melting Approach for Scanning Beta Globin Gene Point Mutations in the Greek and Other Mediterranean Populations

**DOI:** 10.1371/journal.pone.0157393

**Published:** 2016-06-28

**Authors:** Christos Chassanidis, Effrossyni Boutou, Ersi Voskaridou, Angeliki Balassopoulou

**Affiliations:** 1 Molecular Genetics Laboratory, Thalassaemia and Hemoglobinopathies Center, Laiko General Hospital, Athens, Greece; 2 Thalassaemia and Hemoglobinopathies Center, Laiko General Hospital, Athens, Greece; Aristotle University of Thessaloniki, GREECE

## Abstract

Beta-thalassaemia is one of the most common autosomal recessive disorders worldwide. The disease’s high incidence, which is observed in the broader Mediterranean area has led to the establishment of molecular diagnostics’ assays to prevent affected births. Therefore, the development of a reliable, cost-effective and rapid scanning method for β globin gene point mutations, easily adapted to a routine laboratory, is absolutely essential. Here, we describe, for the first time, the development of a High-Resolution Melting Analysis (HRMA) approach, suitable for scanning the particularly heterogeneous beta globin gene mutations present in the Greek population, and thus adaptable to the Mediterranean and other areas where these mutations have been identified. Within this context, β globin gene regions containing mutations frequently identified in the Greek population were divided in ten overlapping amplicons. Our reactions’ setup allowed for the simultaneous amplification of multiple primer sets and partial multiplexing, thereby resulting in significant reduction of the experimental time. DNA samples from β-thalassaemia patients/carriers with defined genotypes were tested. Distinct genotypes displayed distinguishable melting curves, enabling accurate detection of mutations. The described HRMA can be adapted to a high-throughput level. It represents a rapid, simple, cost-effective, reliable, highly feasible and sensitive method for β-thalassaemia gene scanning.

## Introduction

Inherited haemoglobin disorders (HD) are the most frequent monogenic diseases worldwide. Seven per cent of the world’s population is estimated to carry a mutation in the beta (β) globin gene (HBB) (GenBankgenomic reference sequence NG_000007.3), in a heterozygous state (carrier), while thousands of newborns are affected by a severe HD. HDs comprise the α and β thalassaemias, sickle-cell disease (SCD) and other haemoglobinopathies [1–3]. HDs were initially prevalent across the malaria zone (including the Mediterranean, Middle East and Balkan Peninsula areas);yet nowadays, due to migration, they are practically present all across the globe, according to reports from the Thalassaemia International Federation (TIF) [IthaMaps, IthaNet portal,(http://www.ithanet.eu/db/ithamaps)].

Beta–thalassaemia (β-thalassaemia), along with sickle-cell disease (SCD), are the most frequent genetic disorders in Greece, where the mean frequency trait is estimated at 7–8%, and 1–2%, respectively [4–6]. Based on these data, the total number of affected newborns would be expected to be about 150 per year [6]. Hence, within the National Prevention Program framework, a strategy for the prevention of the disease was established almost forty years ago. Through prenatal diagnosis and general population carrier screening, the implementation of this program brought about a dramatic decrease (<10 annually) in the number of affected newborns [6]. The main methodological approach of the above program consists of a combination of haematological analysis, Hb electrophoresis and molecular diagnostics[4–6].

The majority of β-thalassaemia genetic defects are point mutations of various frequencies in distinct ethnic populations [7,8]. In Greece, the most prevalent molecular defect is the IVS I-110 (G>A) mutation, followed by CD39 (C>T), IVS I-1 (G>A) and IVSI-6(C>T) [9,10]. Moreover, due to its high heterogeneity, the range of beta globin mutations in the Greek population can serve as a model, which allows for screening of the most representative β-thalassaemia mutations in the neighboring countries [IthaMaps, IthaNetportal,(http://www.ithanet.eu/db/ithamaps))]. Importantly, because of recent migration flows, a significant percentage of the Mediterranean and Middle East inhabitants have moved to the Central and Northern Europe, thereby increasing the incidence of thalassaemia in this part of Europe as well.

Within this context, implementing a reliable, cost effective and rapid scanning analysis for the HBB gene is required, in order to obtain trustworthy results, which are essential for Prenatal Diagnosis (PND) and personalized therapeutic protocols for the above-mentioned populations. Denaturing gradient gel electrophoresis (DGGE) analysis, denaturing high performance liquid chromatography (DHPLC), single strand conformational polymorphism analysis (SSCP) and Sanger Sequencing are scanning/screening methods that have been applied thus far, but although highly reliable, they are laborious and time consuming. On the other hand, high-resolution melting analysis (HRMA), which represents the next generation of mutation-scanning technology, can easily be adapted in a diagnostic laboratory. The method relies on the ability to distinguish alterations in composition, length, GC content, or strand complementarity of DNA sequences. A PCR-based amplification of the region of interest (amplicon), in the presence of a dsDNA binding dye, precedes HRM analysis. Amplification is followed by a high-resolution melting step, using instrumentation that captures numerous fluorescent data points per temperature change, in a highly precise manner. When dsDNA dissociates (or melts) into single strands, the dye is released, causing a reduction in fluorescence. The result is a melting curve profile that is characteristic of the amplicon’s primary structure. Overall, based on its properties, HRMA emerges as a promising tool for scanning/genotyping in diagnostic laboratories [7, 11–13].

Over the last years, several studies have been conducted using HRM analysis, aiming at rapidly detecting various genetic alterations, including beta-thalassaemia mutations [7, 14–18]. Here, we describe, for the first time to our knowledge, the development of a β-thalassaemia specific HRMA approach, adapted to the Greek population mutation range, in order to replace the currently applied methodology. Our approach focuses not only on creating a rapid and reliable mutation detection assay, but also on providing a cost-effective diagnostic tool. Towards this goal, we designed a simultaneous amplification of multiple primer sets and partial multiplexing real-time PCR, based on identical PCR conditions for all examined amplicons, thereby reducing the number of reactions required to scan all selected regions of the beta globin gene.

## Materials and Methods

### DNA samples

DNA samples (n = 198) that were used for the initial validation of this retrospective study originated either from patients [thalassaemia major (TM), thalassaemia intermedia (TI)], or from previously diagnosedβ-thalassaemia carriers and fetuses. This study is part of the ThalaMoSS project (FP7, THALAssaemia Modular Stratification System for personalized therapy of β-thalassaemia, 306201), and has been approved by the Ethics Committee of Laiko General Hospital, Athens, Greece (No 1551/21-12-2012). Patients’ samples were provided by the Thalassaemia and Hemoglobinopathies Center’s Clinic of Laiko General Hospital. The classification of patients as thalassaemia major (TM) or thalassaemia intermedia (TI) was done according to the clinical database of Thalassaemias’ Center Clinical database. Importantly, this database includes all demographic characteristics. A written informed consent has been obtained from all participants, including parents of minors. Blood sampling was carried out from 2013 to 2015. At least 8 different samples, heterozygous for frequent mutations, were used for the analysis (Table 1), while about 2 to 4 different samples, heterozygous for rare mutations/variations, were also included (Tables 2 and 3). DNA was extracted from peripheral whole blood, amniotic fluid (AF) or chorionic villus sampling (CVS) (Maxwell®16 Blood DNA Purification Kit, Maxwell®16 Cell LEV DNA Purification Kit and Maxwell®16 Tissue DNA Purification Kit, respectively), using the Maxwell®16 Instrument (Promega Corp., Madison, WI, USA) according to the manufacturer’s instructions. Genomic DNA was eluted to a concentration of approximately 30ng/μl (blood) and 10ng/μl (CVS, AF). DNA concentration was measured using the Nano Photometer™ P-Class 330 (Implen, Munchen, DE).

**Table 1:** Distribution and relative frequencies of the common β-thalassaemia mutations in the Greek population and the respective HRM fragment.

Mutation		Ref. Seq. (dbSNP)	Frequency % Boussiou et al. (2008)	HRM Fragment
**IVSI-110, G>A**	β+	rs35004220	42.1	TH3
**CD39, C>T**	β0	rs11549407	18.8	ΤΗ3
**IVSI-1, G>A**	β0	rs33971440	12.8	ΤΗ2Β
**IVS I-6, T>C**	β+	rs35724775	8.1	ΤΗ2Β
**IVS II-745, C>G**	β+	rs34690599	6.3	ΤΗ1, ΤΗ6–7
**IVS II-1, G>A**	β0	rs33945777	3.3	ΤΗ4
**CD6,–A**	β0	rs63749819	1.7	ΤΗ2Α
**-101, C>T**	β++	rs63751208	1.6	ΤΗ1
**-87, C>G**	β+	rs33941377	1.0	ΤΗ1
**CD5,–CT**	β0	rs34889882	0.8	ΤΗ2Α
**CD8,–AA**	β0	rs35497102	0.8	ΤΗ2Α
**Rare**			2.7	

**Table 2:** Rare β-thalassaemia mutations in the Greek population (frequency <0,5% each) and their respective HRM fragments.

Mutation		Ref. Seq. (dbSNP)	HRM Fragment
**-86, C>A**	β++	rs33994806	ΤΗ1
**-30, T>A**	β+	rs33980857	ΤΗ1
**Cap+1, A>C**	β++	rs34305195	TH1
**IVS I-2, T>C**	β0	rs33956879	ΤΗ2B
**IVS I-5, G>A**	β+	rs33915217	ΤΗ2Β
**IVS I-116, T>G**	β0	rs35456885	ΤΗ3
**IVS II-848, C>A**	β+	rs33913413	ΤΗ6–7
**Cap+1480, C>G**	β++	rs34809925	ΤΗ5
**Cap+1570, T>C**	β++	rs34029390	ΤΗ5
**PolyA, AATAAA>AATGAA**	β+	rs63751128	ΤΗ5

**Table 3:** β-thalassaemia variations in the Greek population and the respective HRM fragment.

Variation		Ref. Seq.(dbSNP)	HRM Fragment
**-340, C>T**	SNP	rs10742583	XPro
**-223, T>C**	SNP	rs139703273	XPro
**-190, G>A**	SNP	rs753344875	XPro
**-126, C>T**	SNP	N/A	ΤΗ1
**Cap+20, C>T**	SNP	rs63750628	TH1
**CD2, CAC>CAT**	SNP	rs35906307	TH2A
**IVS II-16, C>G**	SNP	rs10768683	TH4
**IVS II-74, T>G**	SNP	rs7480526	-
**IVS II-666, C>T**	SNP	rs1609812	TH6-7

### Primers

**Fig 1:**
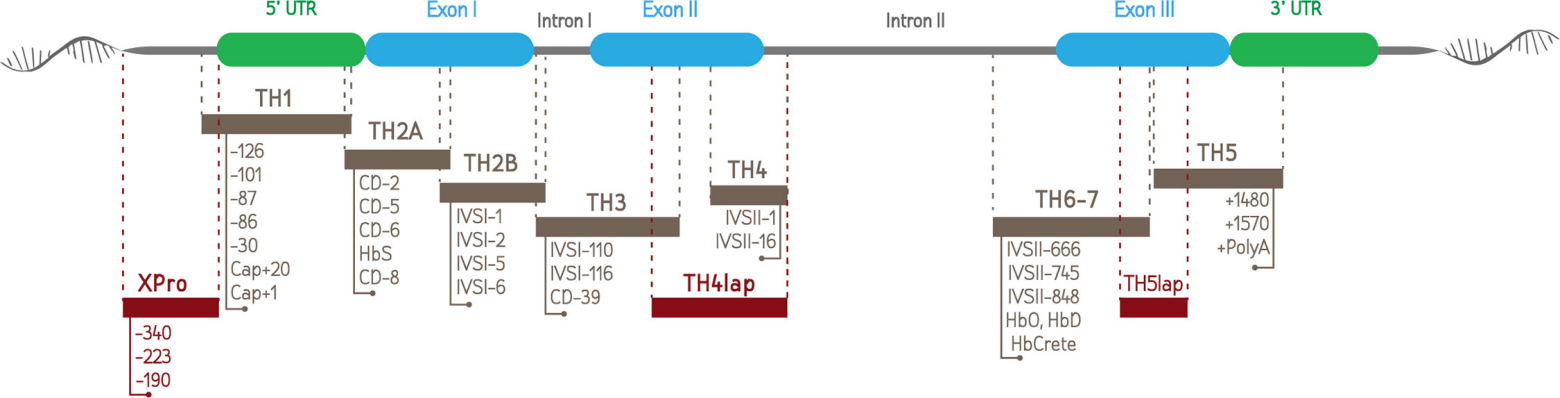
Schematic illustration of the common Greek β-thalassaemia mutations and respective HRMA amplicons.

Specific sets of primers were designed using the Primer 3 and Oligo Analyzer 3.1 (Integrated DNA Technologies) software (NCBI Reference Sequence: NG_000007.3), in order to cover ten regions of the HBB gene containing the hitherto reported mutations/variations of the Greek population (Tables 1, 2, 3 and 4 & Fig 1). Primers were designed so as to have similar Tm. Sequences are shown in Tables 5 and 6. In addition, all ten different and overlapping amplicons were designed and subsequently tested in silico, using the PrimoMelt, WinMelt and uMeltsoftwares, so as to examine the melting properties. Based on the in silico results, certain amplicons were redesigned in an attempt to obtain a unique melting domain, whenever possible. All primers were synthesized and purchased from VBC-Biotech (Vienna, AUT).

**Table 4:** Mutations of frequent haemoglobinopathies in the Greek population and the respective HRM fragment.

Mutation	Ref. Seq. (dbSNP)	HRM Fragment	
**CD6, GAG>GTG, (HbS)**	rs334	TH2A	β chain
**CD 121, GAA>AAA, (Hb O-Arab)**	rs33946267	TH6-7	β chain
**CD 121, GAA>CAA, (Hb D-Punjab)**	rs33946267	TH6-7	β chain
**CD 129, GCC>CCC, (Hb Crete)**	rs35939430	TH6-7	β chain

**Table 5:** Sequences of primers used for HRM analysis of the HBB gene mutations in the Greek population.

Fragment	Region of Detection	Sequence	Length of amplicon	NG_000007.3 (nt)	Annealing Temp. (°C)
**TH1**	**Promoter**	TH1F, 5'- CAGAAGAGCCAAGGACAGGT-3'	197 bp	70.398➜70.595	57
		TH1R, 5’-TGGTGTCTGTTTGAGGTTGC-3'			
**TH2A**	**Exon I**	TH2AF, 5'-TCACTAGCAACCTCAAACAGACA-3'	100 bp	70.570➜70.670	57
		TH2AR, 5'-CACCAACTTCATCCACGTTC-3'			
**TH2B**	**Exon I& Intron I**	TH2BF, 5'-CAAGGT GAACGTGGATGAAG-3'	146 bp	70.645➜70.791	57
		TH2BR, 5'-GGCAGAGAGAGTCAGTGCCTA-3'			
**TH3**	**Intron I & Exon II**	TH3F, 5'-GAAACTGGGCATGTGGAGAC-3'	180 bp	70.728➜70.908	57
		TH3R, 5'-TCACCTTAGGGTTGCCCATA-3'			
**TH4**	**Exon II & Intron II**	NTH4, 5’-AGCTG CACTGTGACA AGCTGCAC-3’	83 bp	70.996➜71.079	57
		TH4lapR, 5-’ AAGAAGGGGAAAGAAAACATCA-3’			
**TH6-7**	**Intron II & Exon III**	TH6F, 5'- TCTGGGTTAAGGCAATAGCAA-3‘	304 bp	71.681➜71.985	57
		TH7R, 5'- ACCAGCCACCACTTTCTGAT-3’			
**TH5**	**Exon III & 3’-UTR**	TH5AF, 5’-CCTGGCCCACAAGTATCACTA-3’	217 bp	71.997➜72.214	57
		TH5bRGC, 5’-gcggggcgggcggcAATGCACTGACCTCCCACAT-3’			

**Table 6:** Sequences of primers used for scanning (HRMA) of additional regions of the HBB gene in the Greek population.

Fragment	Region of Detection	Sequence	Length of amplicon	NG_000007.3 (nt)	Annealing Temp. (°C)
**XPro**	**Promoter**	XProF, 5’-ACCGAGGTAGAGTTTTCATCCA-3’	378 bp	70.074➜70.452	57
		XProNR, 5’-GCTCCACAGGGTGAGGTCTA-3’			
**TH4LAP**	**Exon II & Intron II**	TH4lapF, 5’-TTGAGTCCTTTGGGGATCTG-3’	227 bp	70.852➜71.079	57
		TH4lapR, 5’-AAGAAGGGGAAAGAAAACATCA 3’			
**TH5LAP**	**Exon III**	TH5lapF, 5'- CAAAGAATTCACCCCACCAG-3‘	110bp	71.934➜72.044	57
		TH5lapRGC, 5-'CGGGCGGCAGAAATTGGACAGCAAGAAAGC-3'			

### PCR reactions & High-Resolution Melting Analysis

PCR reactions were performed in duplicates (20μL final volume) in 96-well reaction plates. LightCycler® 480 High-Resolution Melting Master Mix (Roche Diagnostics), which contains the stoichiometric fluorescent dye ResoLight, FastStartTaq DNA polymerase and all four deoxynucleotides, was used according to the manufacturer’s instructions. Thirty ng of genomic DNA, 2,5mM final concentration of each primer and 2.5mM MgCl_2_ were used per reaction. DNA samples with previously defined HBB genotypes were used either as wild type or as positive controls. PCR conditions (e.g. annealing temperature of 57⁰C) were designed so as to be identical for all amplicons, thus allowing for partial multiplexing (i.e, TH2B-3, TH1-6-7 and Fig 1). Two mM of each primer were used in all multiplex PCR reactions.

The HRM assays were performed on the LightCycler® 480 Instrument (Roche Diagnostics, Mannheim, DE), which is provided with the LightCycler® 480 Gene Scanning Software Version 1.0 (Roche Diagnostics, Mannheim, DE). For Resolight detection, the SYBR Green I filter (533 nm) was selected. Initial denaturation–activation step was set at 95°C for 10min, followed by a 45-cycle program [denaturation, 95°C for 15s; annealing, touchdown 62°C to 57°C (with 0,5°C step size and 1 cycle step delay) for 15s; and elongation, 72°C for 15s with parallel fluorescence reading; acquisition mode, single]. Touchdown PCR enhanced the accuracy of priming and amplification. The subsequent melting program included three steps: denaturation at 95°C for 1 min, renaturation at 40°C for 1 min, and then melting from 70°C to 95°C, followed by 25 acquisitions per °C.

### Gene scanning

Melting curve analysis was performed using the Gene Scanning Software. Normalization of melting curves was the first step. Subsequently, the temperature boundaries were set (pre- and post-melt normalization regions, equaling to 100% of the initial fluorescence and to 0% of the remaining fluorescence after the amplicons DNA dissociation). During the second step, the temperature axis of the normalized melting curves was shifted to the point where the entire dsDNA is completely denatured. Finally, through the difference plot, differences in melting curve shapes were analyzed by subtracting each amplicon’s curve from the control’s curve. This further enabled the clustering of samples into groups. A wild type DNA sample is always used to generate the baseline curve. Identical difference plot curves between duplicates are indicative of the method’s reproducibility. Confirmation of the obtained results, was performed by either DGGE analysis and/or Sanger sequencing, when necessary. Positive (heterozygous and homozygous samples for a specific mutation, when available) along with negative controls (DNA-free blanks) were included in each PCR reaction. The entire experiment, PCR reaction and HRM analysis, took place in a closed tube system and was completed in about ninety minutes.

## Results

All different forms (heterozygous or homozygous) for each distinct mutation/variation were clearly distinguishable in the HRM analysis, as they exhibit completely different melting curves. Moreover, the majority of the lesions were also distinguishable in heterozygosity, compound or not. In a few cases (they will be extensively discussed below), where similar melting curves were observed, haematological and electrophoretic indices denoted that further analysis by means of an alternative method is required. False-positive and false-negative results were not observed.

### Results of the HBB gene HRM Analysis

#### Promoter region analysis (amplicon TH1)

**Fig 2:**
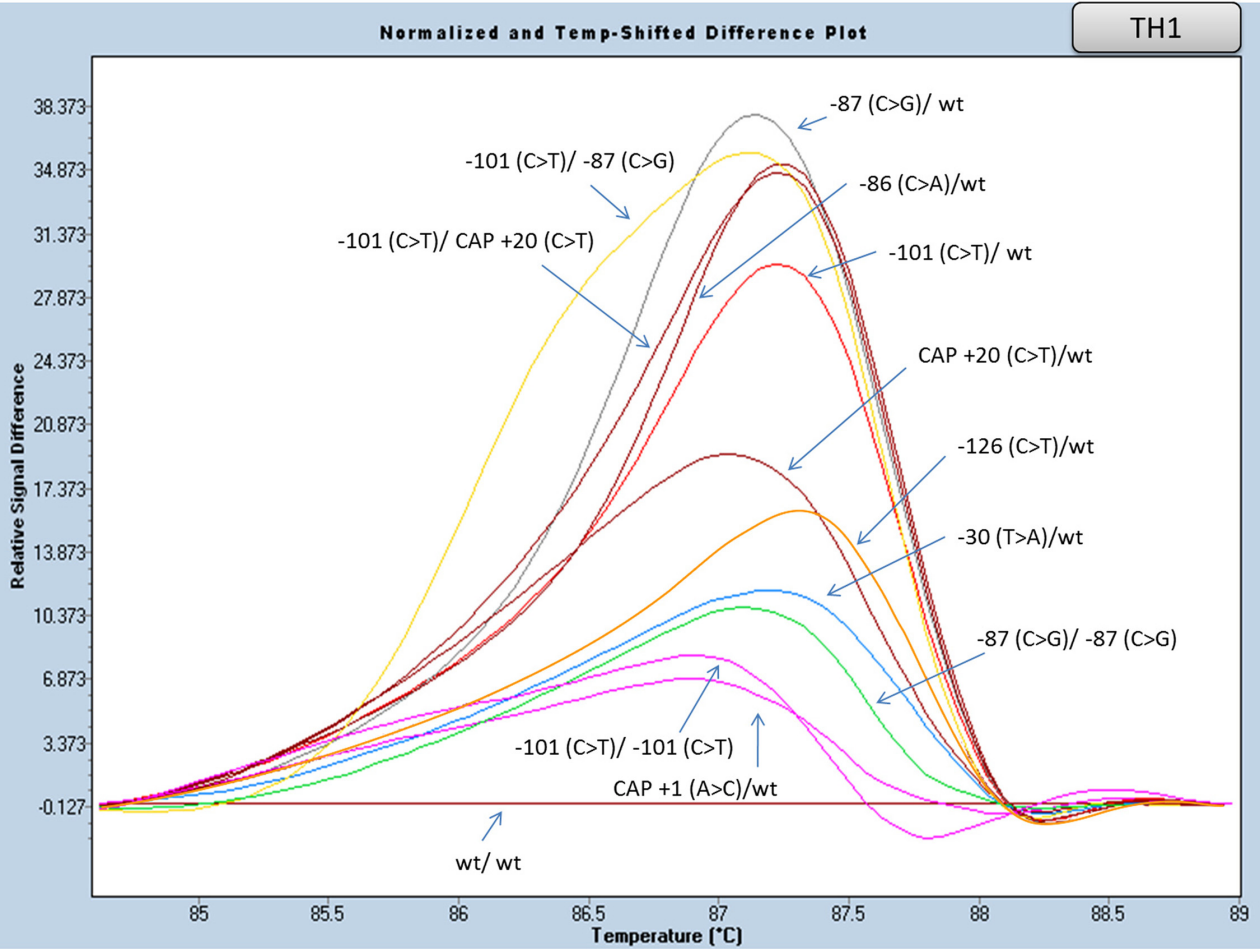
HRM analysis, of HBB gene mutations in the promoter region (amplicon TH1). Normalized and temp-shifted difference plot of various combinations of different mutations/variations (homozygous, heterozygous or compound heterozygous).

The promoter region was analyzed by one amplicon (TH1, Table 5). As shown in Fig 2 and S1 Fig, distinct curves were obtained in all examined mutations and wild type controls. All possible combinations (heterozygous, homozygous and/or compound heterozygous) of the Greek population frequent mutations, which are located in this region, namely, -101 (C>T), -87 (C>G) and CAP +20 (C>T) [polymorphism associated to IVS II-745 (C>G) mutation], are clearly distinguishable.

#### Exon I and flanking region of Intron I analysis (amplicons TH2A and TH2B)

**Fig 3:**
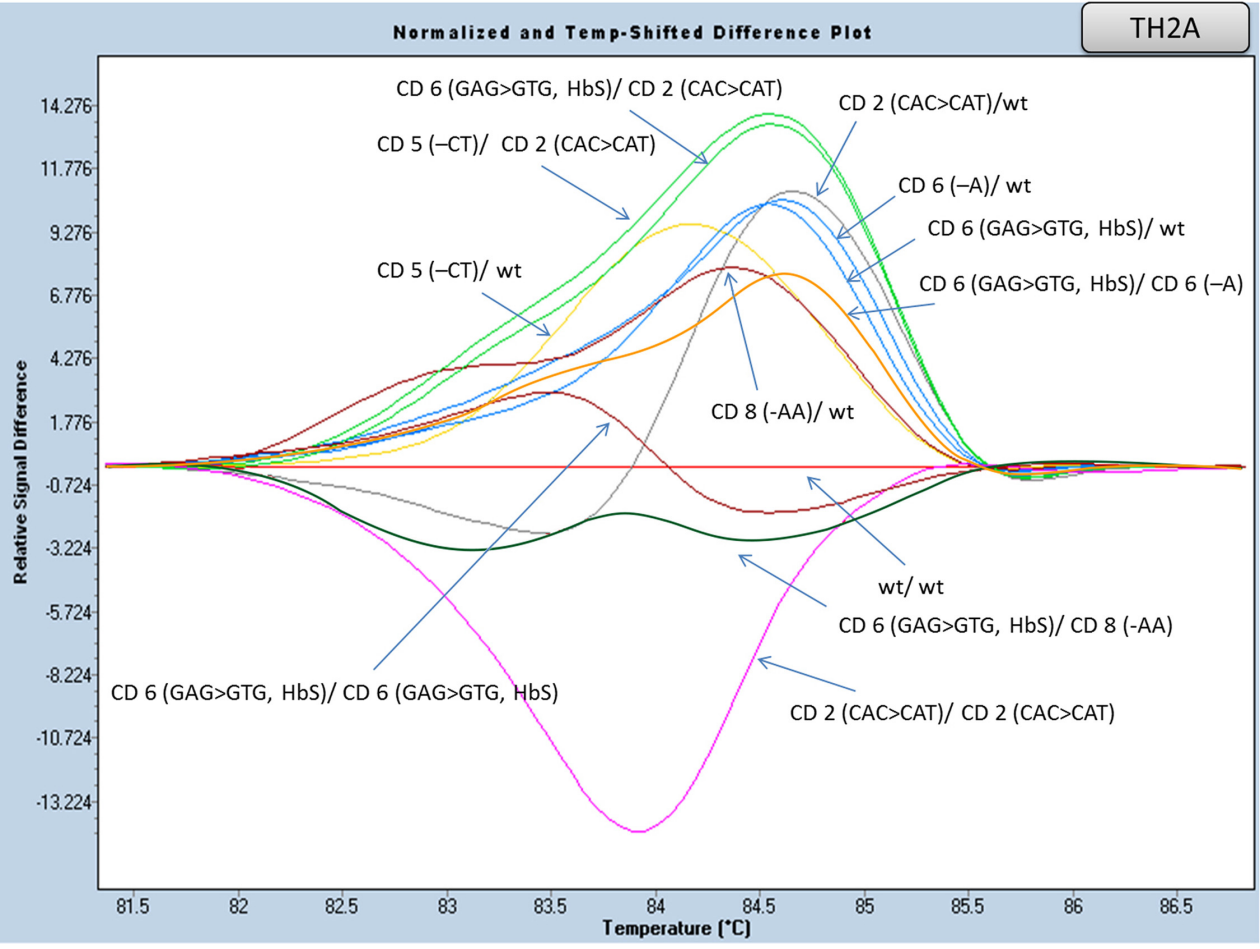
HRM analysis, of the HBB gene mutations in exon I (amplicon TH2A). Normalized and temp-shifted difference plot of various combinations of different mutations/variations (homozygous, heterozygous or compound heterozygous).

**Fig 4:**
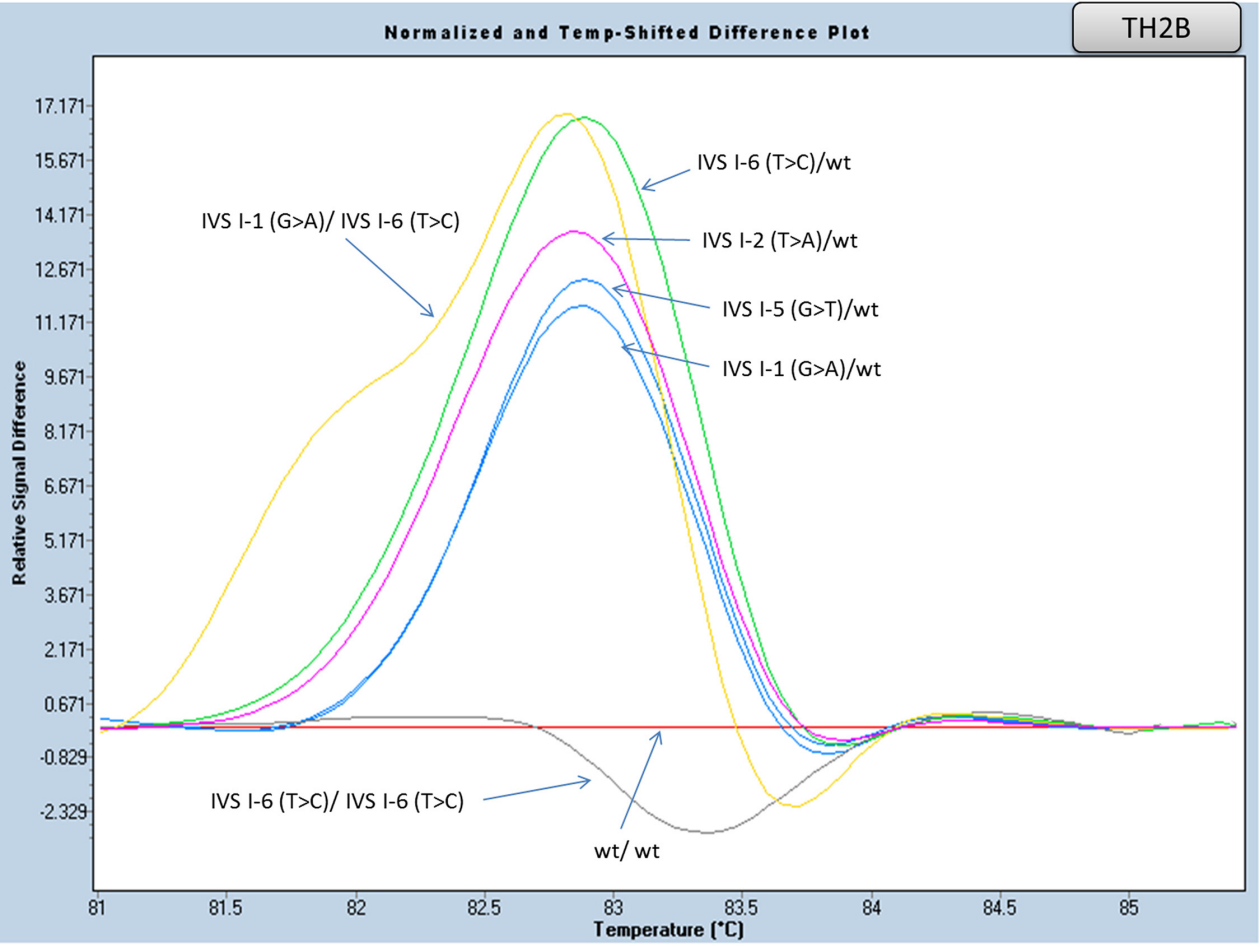
HRM analysis, of the HBB gene mutations in exon I and intron I (amplicon TH2B). Normalized and temp-shifted difference plot of various combinations of different mutations (homozygous, heterozygous or compound heterozygous).

An initial attempt to scan this part of the gene included a small amplicon (TH2, 180 bp), covering the entire exon I, along with 40bp of the flanking intron I region. However, the presence of the CD2 polymorphism (CAC>CAT, in heterozygous and/or homozygous state) rendered difficult the distinction between different genotypes. The high incidence and strong association (in cis) of the above polymorphism with one of the most frequent mutations in the Greek population located in the same amplicon [IVS I-6 (T>C)] prompted us to design two smaller, overlapping, amplicons (TH2A and TH2B, Table 5), in order to avoid multiple melting curves due to the CD2polymorphism. Heterozygous, homozygous and/or compound heterozygous patterns of the most frequent mutations [IVS I-1 (G>A), IVS I-6 (T>C), CD 6 (-A), CD 8 (-AA), CD 5 (-CT) and CD 6 (GAG>GTG, βS)] located in these amplicons, differ clearly from wild type control samples (Figs 3 and 4, S2 and S3 Figs).

#### Part of Exon II and flanking region of Intron I analysis (amplicon TH3)

**Fig 5:**
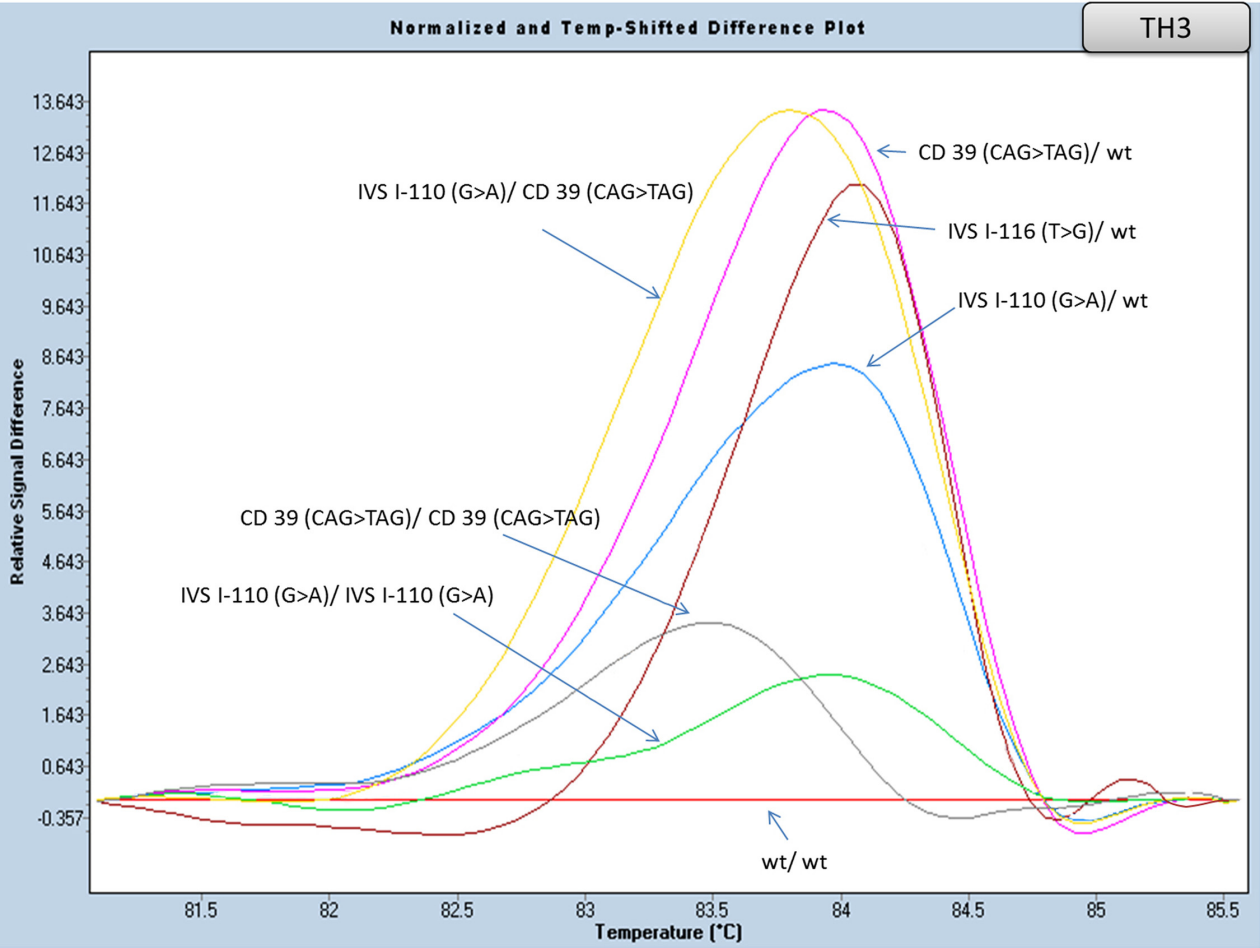
HRM analysis of HBB gene mutations in intron I and exon II (amplicon TH3). Normalized and temp-shifted difference plot of various combinations of different mutations (homozygous, heterozygous or compound heterozygous).

Amplicon TH3 (Table 4) was designed for the detection of the two most frequent Greek β-thalassaemia mutations: IVS I-110 (G>A, 42.5%) and CD 39 (CAG>TAG, 19.6%). All possible genotypes (heterozygous, homozygous and/or compound heterozygous) showed distinct curve profiles, not only between them, but when compared to the wild type as well (Fig 5 and S4 Fig).

#### Part of Exon II and flanking region of Intron II analysis (amplicon TH4)

**Fig 6:**
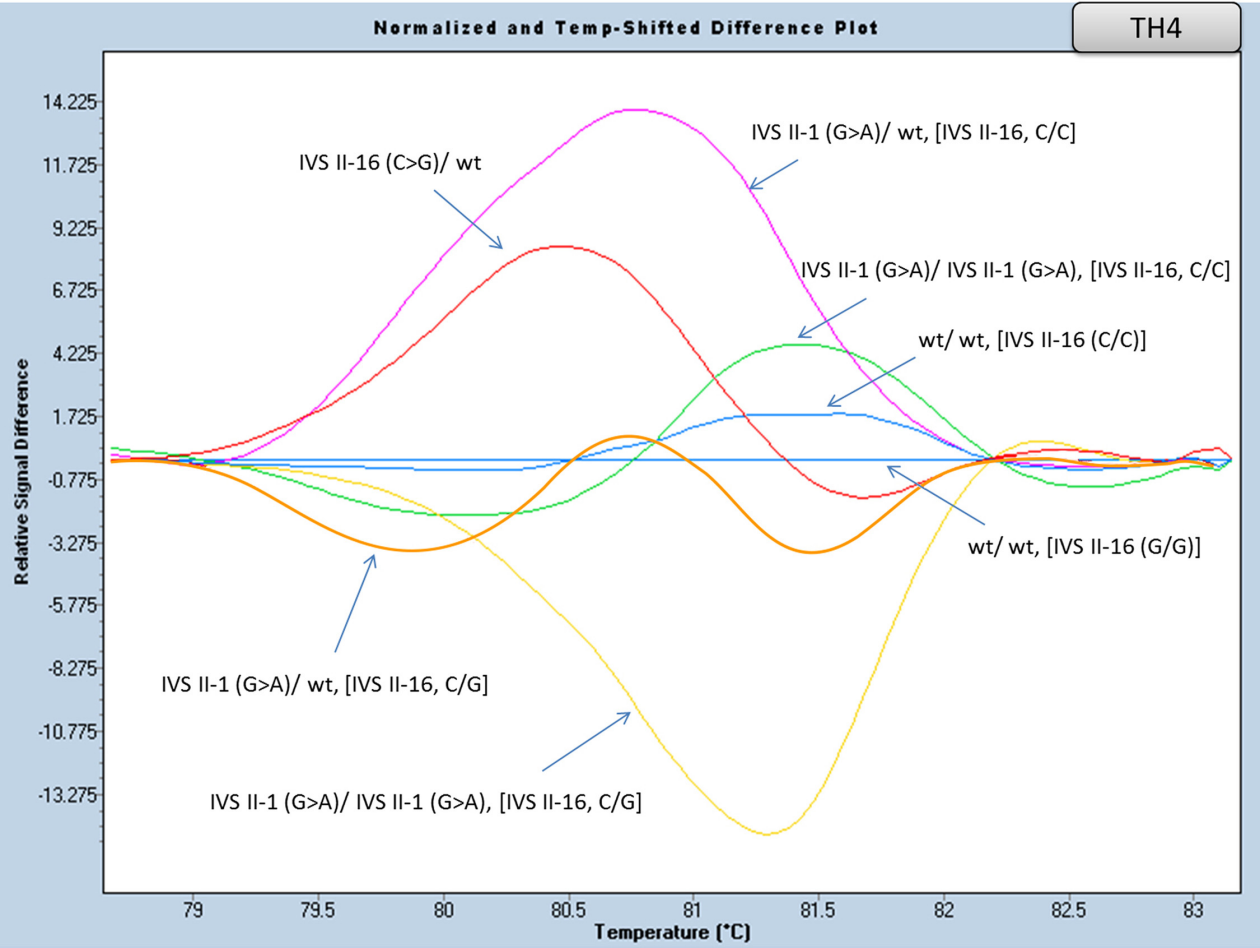
HRM analysis, of the HBB gene mutations in exon II and intron II (amplicon TH4). Normalized and temp-shifted difference plot of various combinations of different mutations/variations (homozygous, heterozygous or compound heterozygous).

A small amplicon (TH4, Table 5) was specifically designed for the detection of the IVS II-1 (G>A, 2.0%) mutation. Due to the presence of two quite frequent polymorphisms in its vicinity, IVS II-16 (G>C) and IVS II-74 (T>G), a reverse primer was designed so as to bind between these two polymorphisms, in order to avoid interference with polymorphism IVS II-74. The PCR product was an extremely small amplicon, but nonetheless led to a distinctive differentiation plot, as shown in Fig 6 and S5 Fig.

#### Part of Exon III and flanking region of intron II analysis (amplicon TH6-7)

**Fig 7:**
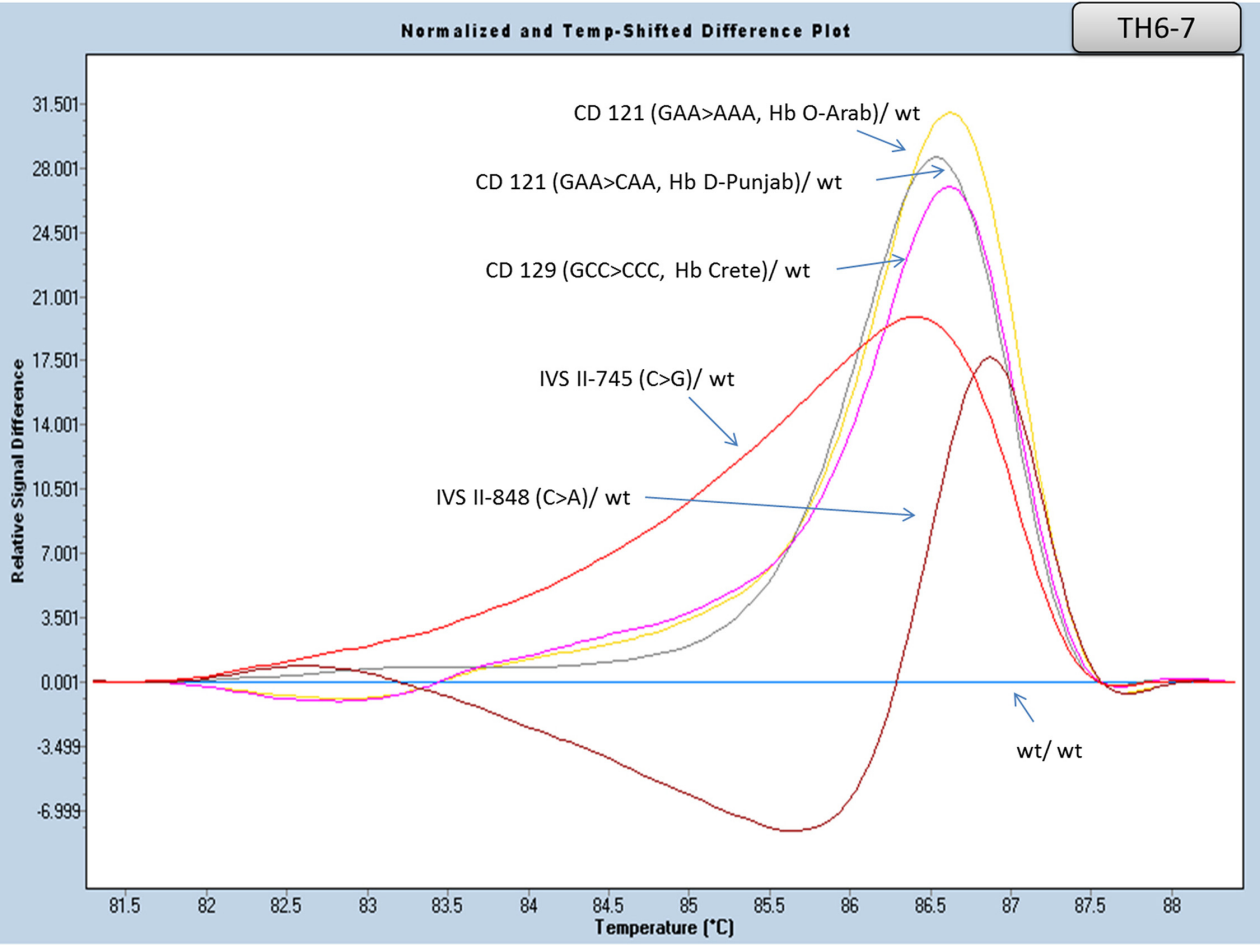
HRM analysis, of the HBB gene mutations in intron II and exon III (amplicon TH6-7). Normalized and temp-shifted difference plot of different mutations (heterozygous).

Two amplicons covering a region of approximately 300 bp (TH6, TH7) (including the end of intron II and the beginning of exon III) were used for this analysis. Both amplicons yielded reliable results when analyzed separately; Equally good results were also observed when analyzed in one merged amplicon (using primers TH6F and TH7R). All heterozygous genotypes regarding the Greek population mutations residing in this amplicon [IVS II-745 (C>G), CD 129 (GCC>CCC, Hb Crete), CD 121 (GAA>CAA, Hb D-Punjab), CD 121 (GAA>AAA, Hb O-Arab), IVS II-848 (C>A)], showed differential HRMA results not only between them, but also when compared to the wild type samples as well (Table 5, Fig 7 and S6 Fig).

#### Part of Exon III and part of the flanking 3’UTR region analysis (amplicon TH5)

**Fig 8:**
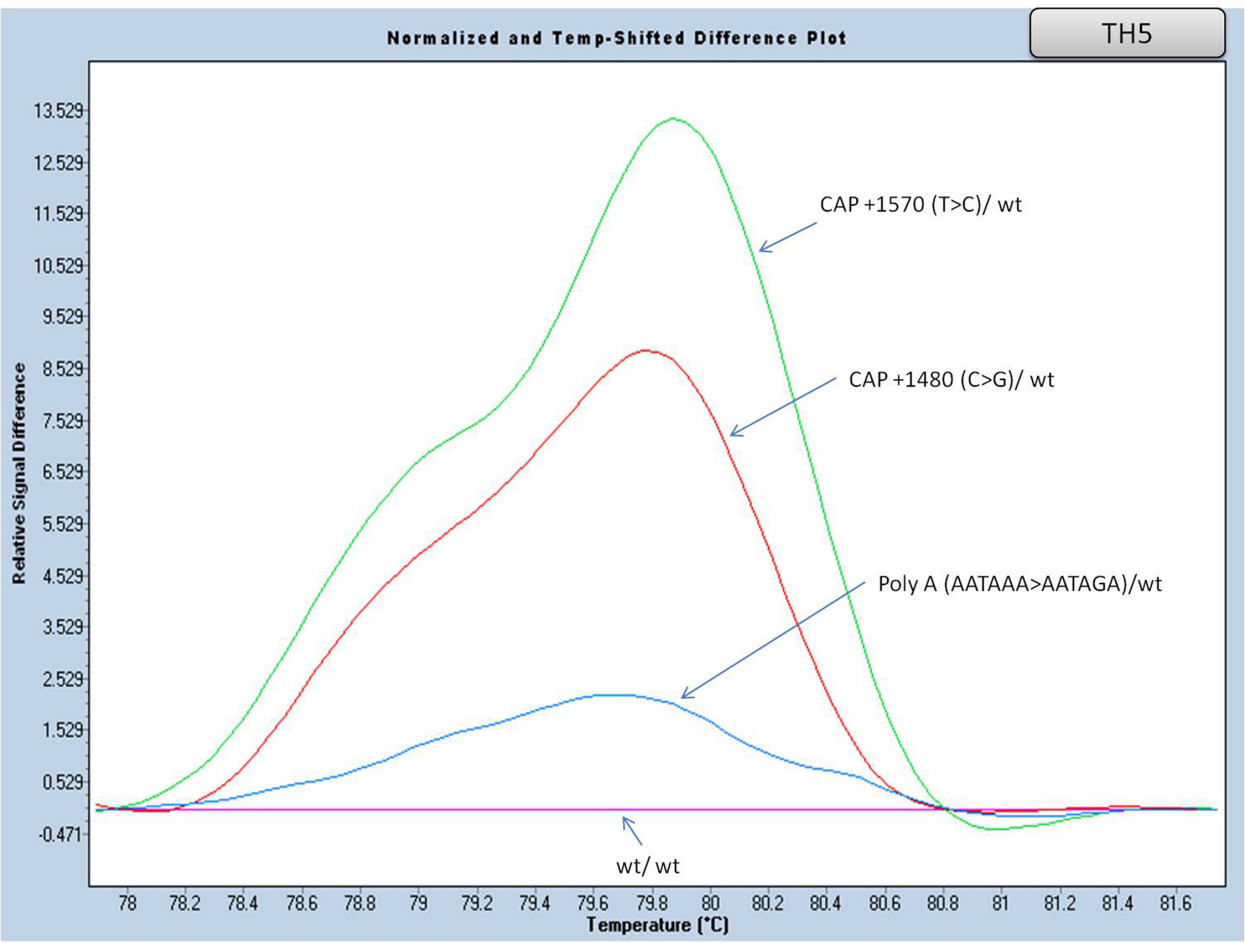
HRM analysis, of the HBB gene mutations in exon III and 3’UTR (amplicon TH5). Normalized and temp-shifted difference plot of different mutations (heterozygous).

Amplicon TH5 (Table 5) covers a region of exon III and the 3’UTR of the β globin gene. In this HRM analysis, differential melting curves were observed in the presence of two mild (β^++^) [CAP +1480 (C>G), CAP +1570 (T>C)] and one β^+^ mutation [Poly A (A>G, AATAAA>AATAGA)], in heterozygosity. The above mutations are found in low frequency in the Greek population (Fig 8 and S7 Fig).

#### Additional HBB gene regions analysis

**Fig 9:**
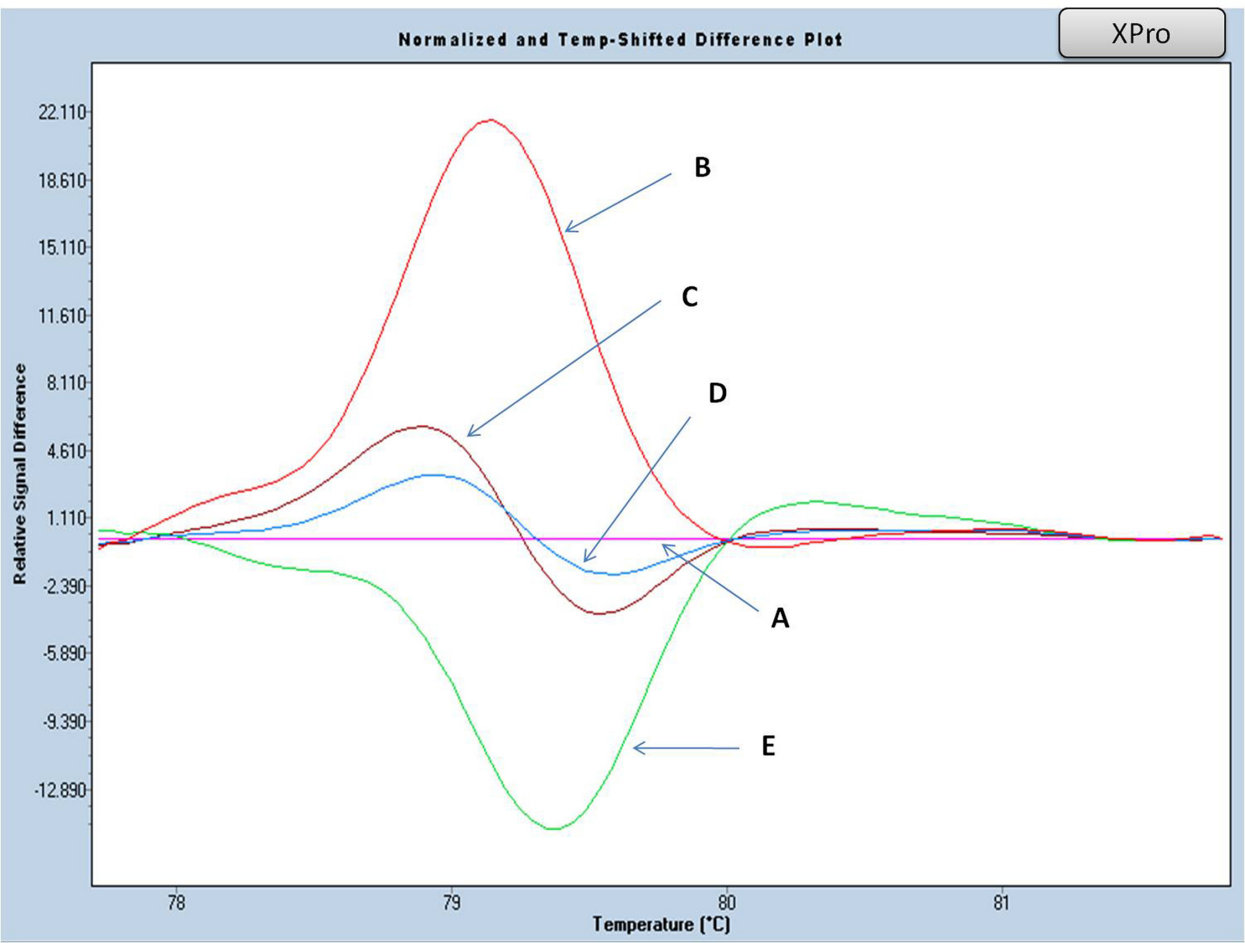
HRM analysis, of 5’UTR mutations of the HBB gene (amplicon XPro). Normalized and temp-shifted difference plot of various combinations (homozygous, heterozygous or compound heterozygous) of three different variations (rs10742583: -340C>T, rs139703273: -223T>C, rs753344875: -190G>A). Genotypes cannot be fully defined in terms of cis or trans inheritance. Homozygous -340 (T) was set as a base curve. A: -340 (T/T), -223 (T/T), -190 (G/G). B: -340 (T/T), -223 (T/T), -190 (G/**A**). C: -340 (T/**C**), -223 (T/T), -190 (G/G). D: -340 (T/T), -223 (T/**C**), -190 (**A**/**A**). E: -340 (T/T), -223 (T/**C**), -190 (G/G).

In order to cover the entire β globin gene sequence, additional amplicons were designed to assay for regions that were not included in the previous design. Although analysis of these regions may not be useful for routine general population screening, since no mutation has thus far been detected in these regions in the Greek population, it may provide a more extensive analysis of undefined genotypes in rare cases. Even if they are present in a thalassaemic carrier or patient phenotype, such cases remain non-diagnosed with regards to the underlying molecular defects, since the previously described scanning methods do not detect any mutations. Our expanded analysis includes amplicon XPro (Table 6, Fig 9 and S8 Fig), which corresponds to the distal promoter region, and amplicons TH4lap and TH5lap, which correspond to 90 bps at the end of exon II (ng000007.3: nt70909-70995) and 10 bps at the end of exon III (ng000007.3: nt71986-71997). The primer sets are shown in Table 6. Unfortunately, this approach could be adequately assessed for amplicons TH4lap and TH5lap, because no reference control sample was available in our laboratory.

#### Multiplexing

**Fig 10:**
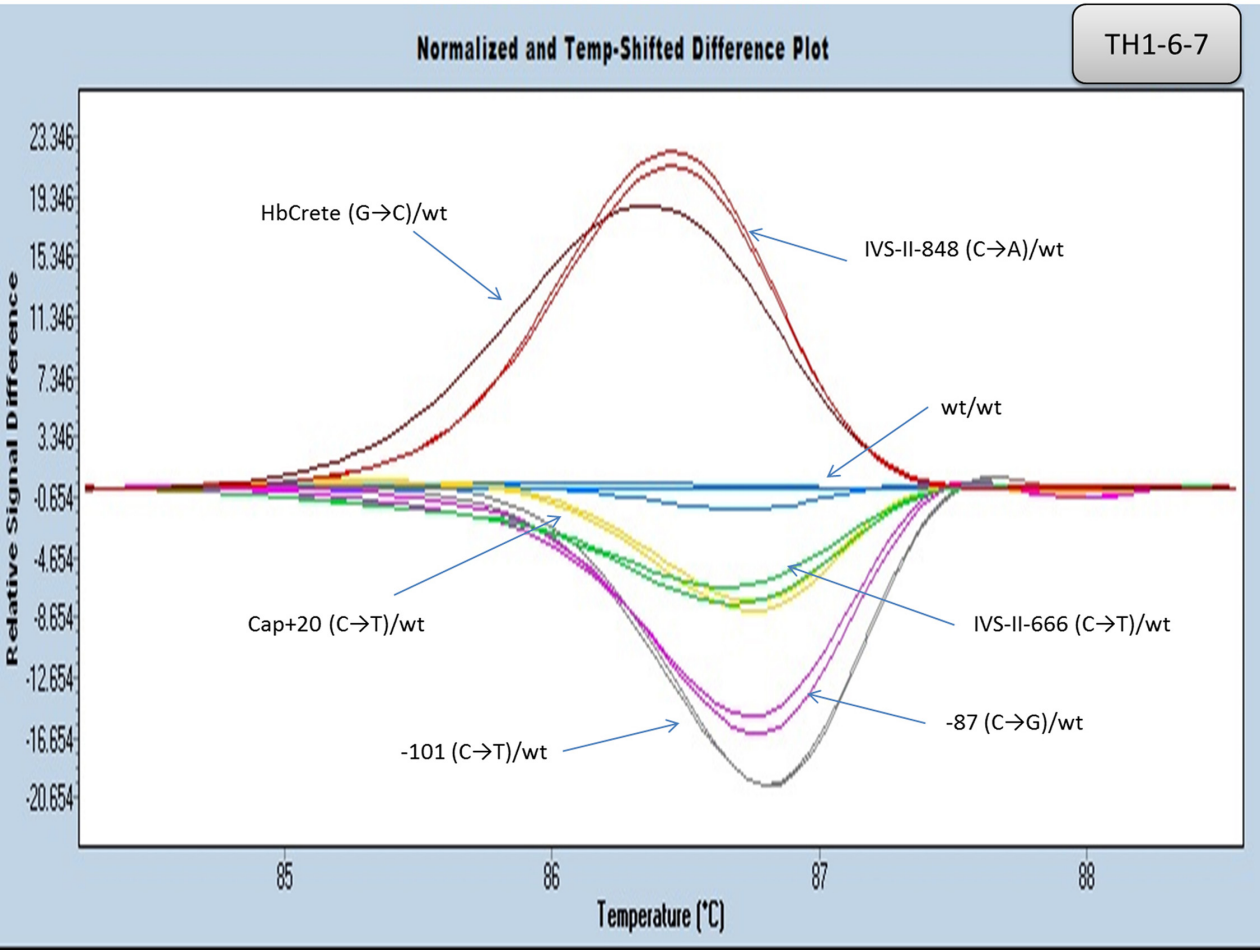
HRM analysis of the HBB gene mutations in promoter, intron II and exon III by multiplex PCR (amplicons TH1-6-7). Normalized and temp-shifted difference plot of different mutations/variations (heterozygous).

**Fig 11:**
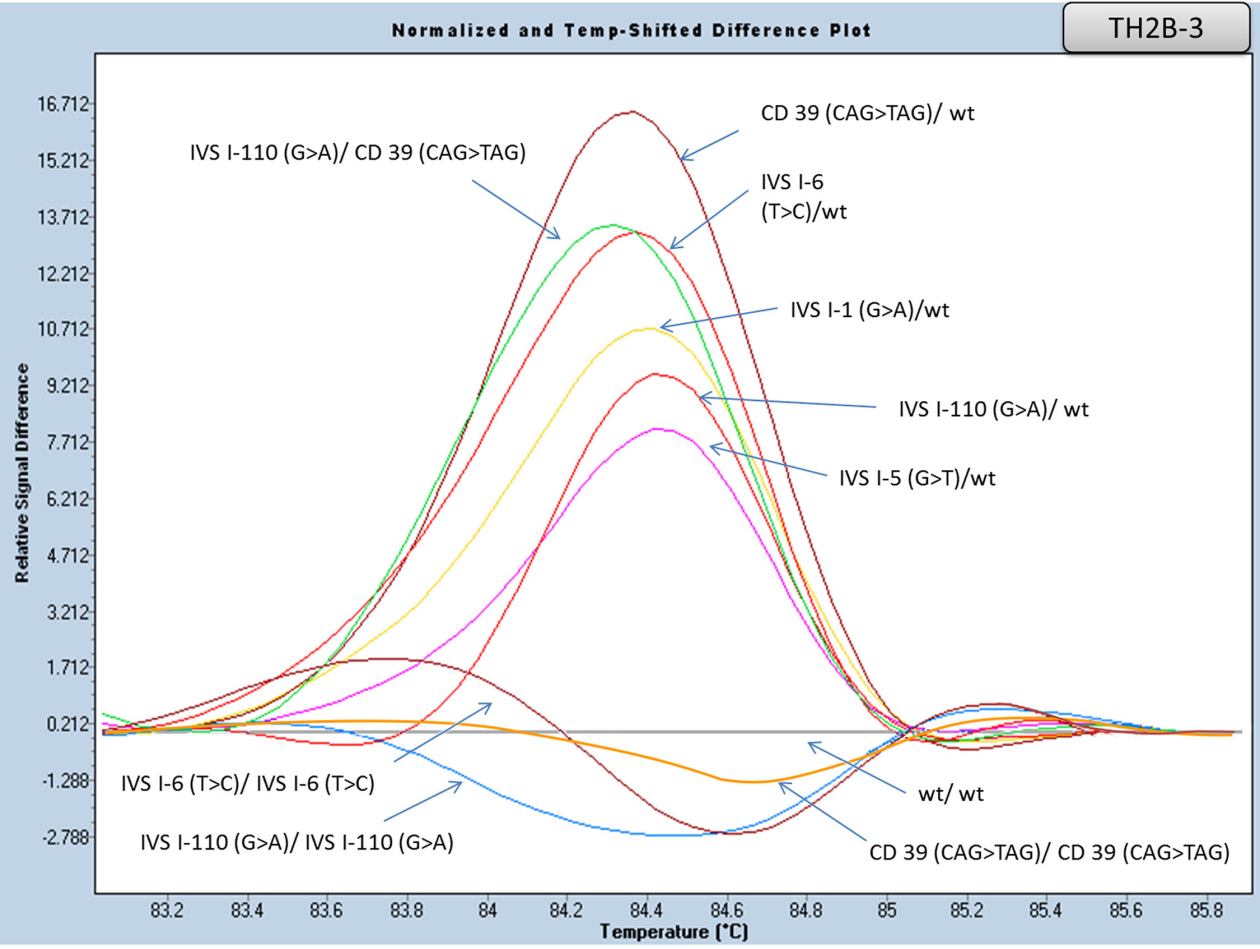
HRM analysis of the HBB gene mutations in exon I, intron I and exon II by multiplex PCR (amplicons TH2B-3). Normalized and temp-shifted difference plot of various combinations of different mutations (homozygous, heterozygous or compound heterozygous).

Aiming at reducing the number of reactions required for a complete routine β globin gene analysis, multiplex PCR reactions of specific amplicons were attempted. As shown in Fig 10 (S9 Fig), amplicons TH1 and TH6-7 were amplified and analyzed simultaneously. In addition, the multiplex reaction performed with primers amplifying both TH2B and TH3 is shown in Fig 11 (S10 Fig). In both cases, distinct melting curves for the tested variants, in both multiplex sets, were observed.

## Discussion

Inherited haemoglobin disorders are among the most frequent hereditary diseases worldwide, being prevalent in the Mediterranean as well as the Asian population. Over the last years, due to migration flows, HD’s incidence has increased in Central and Northern European countries as well [19]. The mean frequency of β-thalassaemia trait in Greece is estimated at 7–8%, but can reach up to 20% in several geographic areas [4,5]. Carrier screening and prenatal diagnosis in couples that are at risk for thalassaemia major (approximately 15000 carrier screening analysis and 250 PND out of 600 expected annually across the country) are currently offered within the context of the National Thalassaemia Prevention Program that was established four decades ago. Importantly, similar programs for the prevention of β-thalassaemia major have also been established in Italy and Cyprus [19]. The United Kingdom and France have also set up a comprehensive national prevention program [19]. Furthermore, programs including antenatal screening have been established in countries such as the Netherlands, Belgium and Germany [19]. Therefore, beta globin genotyping analysis for couples that are at risk is, unquestionably, required for rapid and accurate prenatal diagnosis.

HRM analysis has become the method of choice for mutation scanning/screening for many genetic diseases, replacing previously applied techniques (amplification refractory mutation system-PCR, DGGE analysis, reverse dot-blot hybridization, probe-based real-time PCR). Several studies have been already published for β thalassaemia HRM analysis, since the underlying molecular defects are well characterized [14–18]. All of them have been designed on an ethnic-oriented basis, depending on mutation frequencies of the corresponding ethnic group.

Our study presents the effort to set up–to our knowledge, for the first time–an HRM assay for the rapid detection of HBB gene mutations/variations identified in populations of European and Arabic origin, using, as a model, the highly heterogeneous mutation range of the Greek population.

Amplicons were designed so as to allow for: (*a)* identical amplification conditions for all genomic fragments (same annealing temperature (57⁰C) in particular), thus enabling simultaneous analysis of more than one amplicons; (*b)* distinct multiplexed analysis for frequent mutations (TH2B/TH3), as well as for rare, mild or of low frequency mutations (TH1/6-7). Importantly, polymorphisms that are quite common in the Greek population were taken into consideration, in order to design the primer sets. Within this context, we found that the presence of common Mediterranean polymorphisms did not adversely affect the PCR procedure (failure or low yield).

Our experiment was design so as to detect the four most frequent mutations, IVS I-110/ CD39, IVS I-1/ IVS I-6 (with regards to their frequency range in the Greek population), which account for approximately 84% of all Greek point mutations defined, in amplicons TH3 and TH2B, respectively. In general, all possible combinations (homozygous, heterozygous and compound heterozygous) displayed distinct melting curves between each other and with in comparison to wild type controls. Therefore, the strategy we developed was able to overcome some previously reported difficulties of HRM analysis in distinguishing certain variants in homozygosity, as well as small insertions and deletions [20]. Mutations IVS I-110 (G>A) and CD39 (C>T) (detected in TH3 amplicon), as well as IVS I-1 (G>A) and IVS I-6 (T>C) (detected in TH2B amplicon) can be simultaneously analyzed in one reaction (TH2B/TH3). Each one is clearly distinguished, displaying a specific melting curve (in any combination) when compared to the others or to wild types.

On the contrary, HRM analysis of the TH2A and TH2B amplicons showed only slight differences in the melting curves between CD6 (–A)/wt and CD6 (GAG>GTG, β^S^)/wt, as well as CD5 (–CT)/CD2 (CAC>CAT) and CD6 (GAG>GTG, β^S^)/CD2 (CAC>CAT) genotypes (TH2Aamplicon). While the aforementioned mutations failed to show distinct melting curves, diagnosis can be performed in combination with Hb electrophoresis, where the presence of HbS is clearly detected. The same was true for the IVS I-1 (G>A)/wt and IVS I-5 (G>A)/wt genotype (TH2Bamplicon). In this case, it is the Hb electrophoresis (HbA_2_ percentage) that is indicative, but in any case, further confirmation of the underlying mutation by means of an alternative method is required. Taken together, the above show that the most frequent β globin gene mutations in the Greek population can be screened in a fast and reliable manner, merely by performing HRMA of three amplicons, namely the TH3, TH2A and TH2B.

With regards to the detection of low frequency, rare and mild mutations/variants of the beta globin gene, such as -126 (C>T), -101(C>T), -87 (C>G), -86 (C>A), -30 (T>A), CAP +1 (A>C), IVS II-666 (C>T), IVS II-745 (C>G), IVS II-848 (C>A), CD 121 (GAA>CAA, Hb D-Punjab), CD 121 (GAA>AAA, Hb O-Arab), CD 129 (GCC>CCC, Hb Crete), CAP +1480 (C>G), CAP +1570 (T>C), Poly A (A>G, AATAAA>AATAGA), we designed three amplicons, TH1, TH6-7 and TH5. In the case of TH1, successful detection due to distinctive melting curves was observed for the following mutations in homozygosity: -101(C>T), -87 (C>G), as well as in compound heterozygosity: -101 (C>T)/ -87 (C>G). The -101 (C>T) mutation, is the most frequent β^++^ (mild) one in the Greek population, and is related to a very mild or even normal haematological phenotype. Compound heterozygosity with a β° mutation results in a slightly more severe haematological phenotype, compared to that of the β°heterozygote. No clinical symptoms are observed in this case. Because of this, prenatal diagnosis is not suggested in such cases; therefore the -101 (C>T) mutation detection is useful for appropriate genetic counseling, considering the clinical manifestation of this mutation. The CAP +20 (C>T) polymorphism, in linkage (in cis) with the β° IVS II-745 (C>G) mutation, is also detected in the TH1 amplicon, indicating or further confirming the presence of the IVS II-745 (C>G) mutation. Furthermore, the similar melting curves observed between the following genotypes, -101 (C>T)/ CAP +20 (C>T) and -86 (C>A)/wt as well as -101 (C>T)/ -101 (C>T) and CAP +1 (A>C)/wt, is considered as a problem that can be easily overcome since haematological electrophoretic analysis help to distinguish the above mutations. Analysis of amplicons TH6-7 permits the identification of β-thalassaemia mutations, as well as of mutations causing frequent hemoglobinopathies. In particular, genotype CD 121 (GAA>AAA, Hb O-Arab)/wt is clearly distinct from CD 121 (GAA>CAA, Hb D-Punjab)/wt. This result is an important advantage of the developed HRM approach, since the above haemoglobins cannot be distinguished by HPLC analysis. Moreover, multiplexing of TH1 and TH6-7 amplicons has worked efficiently (Fig 10). Regarding exon III and 3’UTR, the TH5 amplicon reaction was successfully established, detecting three gene mutations (CAP +1570, CAP +1480, Poly A). There is a contradictory issue about CAP +1570: it has been considered either a very mild mutation or even a neutral polymorphism. Recent data has classified CAP +1570 as a β^++^ mutation, thereby rendering its detection valuable for genetic counseling, similarly to the -101 (C>T) case described above [21]. Thus, mutations responsible for mild phenotypes can be detected very rapidly, applying three different PCR reactions (amplicons TH1, TH6-7 and TH5).

Theβ^0^ IVS II-1 (G>A) mutation is detected separately, by analyzing the TH4 amplicon, as described in the Results section. This approach is chosen because of its distant location compared to other frequent mutations in the Greek population. The presence or absence of the IVS II-16 polymorphism, located in the vicinity of the IVS II-1 (G>A) mutation, influenced the melting curve pattern, thereby resulting in different profiles according to the present combination. Quite importantly, almost all melting curve profiles were distinct from those of the corresponding wild type control, except for the homozygous IVS II-16 genotype curve, which coincided with one of the wild type control. Nevertheless, IVS II-16 is considered to be a neutral polymorphism without any clinical significance, and therefore its presence is rather irrelevant for diagnosis.

Finally, extended investigation of the promoter region toward the 5’ UTR led to the identification of a rare variant [22] (one reference only), -190 (G>A), in two samples of the same germline. The -190 (G>A) variant, which is referred as a causative mild mutation in the Ithanet database, is rather a polymorphism, as in our study, it shows complete linkage (in cis) with CD6 (-A). A slight difference between the melting curves of the above samples is due to the co-presence of the -223 T>C polymorphism in one of them (Fig 9). Furthermore, promoter region analysis revealed that, due to its high frequency (10/10 samples homozygosity, 20 chromosomes), the-340 (T) allele seems to be the wild type in the Greek population, instead of the -340 (C), which is used as a reference sequence in HBB databases (ng_000007.3).

Overall, the presented HRMA is stable and reproducible: the same samples, used for internal controls, depicted, after specific normalization, the same characteristic melting curves in the appropriate Tm (S11–S14 Figs). Our results clearly show that HRM analysis may represent an alternative method for the molecular genetic diagnosis of point mutations and small indels. Frequently, large deletions in the β globin gene cluster [common deletions in the Greek population are the δβ-Sicilian type, δβ-Turkish type and δβ-Lepore (0,5–1% in the general population according to our Centre’s unpublished data)] are not detectable through the aforementioned experimental procedure. Moreover, large deletions were never confounding the diagnostic procedure, even in previously used diagnostic methods (DGGE), since hematological indices could clearly lead to deletion detection using gap-PCR method. Some laboratories still use DGGE and SSCP analysis approaches for HBB gene scanning. Application of HRM analysis is a significantly faster, safer and equally reliable method, as it does not require any post-PCR analysis and thus avoids the use of hazardous and polluting chemicals (formamide, ethidium-bromide, acrylamide). Moreover, the cost is relatively similar, especially when multiplexing is implemented. HRM analysis of the PCR products is processed by computing software, based on characteristics such as length, melting domain (GC content) and strength of the fluorescence signal. In addition to known mutations, novel distinct patterns that may correspond to rare or novel variants are indicative of the presence of sequence alterations within the tested amplicon. Samples with such non-defined melting curves, clearly differing from the wild type, require subsequent analysis by Sanger sequencing. Although DNA sequencing could be a method of choice for HBB gene analysis, HRM is preferred thanks to its higher rapidity and cost effectiveness. Next Generation Sequencing (NGS), a rapidly developing method that is already being used in research and in specific diagnostic applications, could be an alternative. Up to date, NGS is difficult to establish in everyday diagnostics, because of its high cost and problems in accurate result interpretation related to aspects of bioinformatics.

Another important issue is that rapid and reliable HBB genotype definition is a crucial step in terms of genetic counseling, especially for pregnancies of advanced gestatiοnal age, where the β-thalassaemia status of one or both partners is not clearly defined by haematological and electrophoretic indices. Such cases concern involvement of iron deficiency anemia (IDA), co-inheritance of δ-thalassaemia and/or borderline haematological and electrophoretic indices.

**Fig 12:**
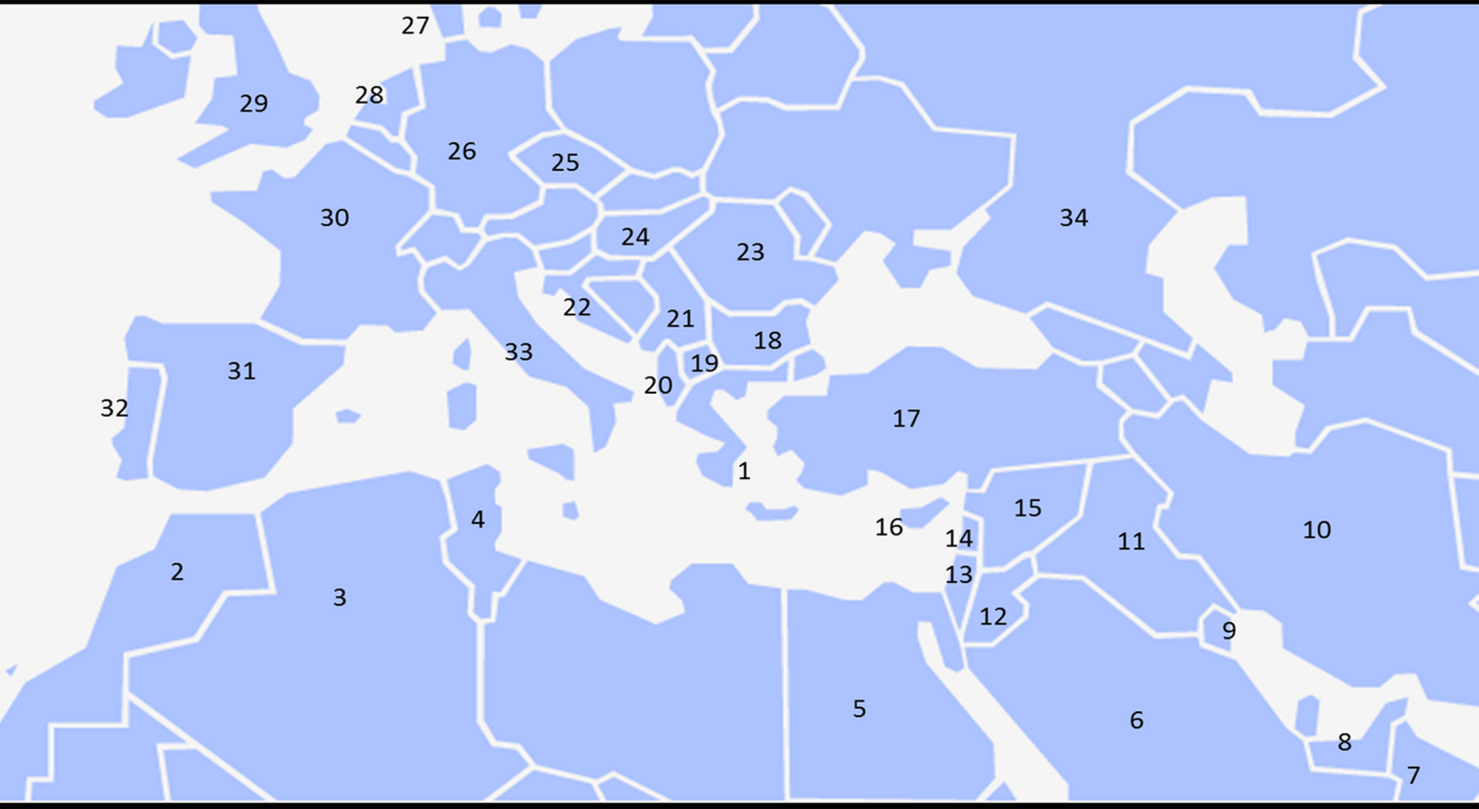
Map of countries where HDs are present, as described in Table 7, sharing a significant percentage of beta globin gene mutations present in the Greek population.

In conclusion, the developed HRM assay represents a rapid (does not require any post-PCR analysis), simple, relatively easy to perform, cost-effective and highly feasible method, amenable to high-throughput systems and capable of efficiently identifying 97.3% of HBB gene mutations prevalent in Greece. It is interesting that this panel covers the mutation spectrum of most Mediterranean countries, although frequencies may differ in some cases. In this context, Table 7 and Fig 12, based on data from IthaMaps (http://www.ithanet.eu/db/ithamaps), clearly show that six of the most common mutations in the Greek population [IVS I-110 (G>A), CD39 (C>T) IVS I-1 (G>A), IVSI-6(C>T), IVS II-745 (C>G), IVS II-1 (G>A)] are present and/or prevalent not only in neighboring countries, but also in Middle East and Central and Northern Europe. The frequent Arabic IVS I-5 (G>C) mutation, although not tested due to lack of control samples, is expected to be distinguishable as predicted by the initial in silico design. The average percentage of total coverage for all countries’ beta globin gene mutation panel reaches up to approximately 77%, arguing that the herein described specific design of beta globin gene HRM analysis could be used for the detection of a significant portion of the mutations found in many European and Asian areas.

**Table 7:** The most common "Greek" mutations of the beta globin gene and their corresponding mutation detection rate for the countries listed (indicated by international short name, map Fig 12) according to the Ithanet database (www.ithanet.eu/db/ithamaps). Average percentage of total coverage for all countries’ beta globin gene mutation panel is 76.6±15.3% (S1 File).

	GR	MA	DZ	TN	EG	SA	OM	AE	KW	IR	IQ	JO	IL	LB	SY	CY	TR
	1	2	3	4	5	6	7	8	9	10	11	12	13	14	15	16	17
**IVSI-110, (G>A)**	**√**	**√**	**√**	**√**	**√**	**√**	**√**	**√**	**√**	**√**	**√**	**√**	**√**	**√**	**√**	**√**	**√**
**CD39, (C>T)**	**√**	**√**	**√**	**√**	**√**	**√**	**√**		**√**	**√**	**√**	**√**	**√**	**√**	**√**	**√**	**√**
**IVSI-1, (G>A)**	**√**	**√**	**√**	**√**	**√**		**√**	**√**	**√**	**√**	**√**		**√**	**√**	**√**	**√**	**√**
**IVS I-6, (T>C)**	**√**	**√**	**√**	**√**	**√**			**√**	**√**	**√**	**√**	**√**	**√**		**√**	**√**	**√**
**IVS II-745, (C>G)**	**√**	**√**	**√**	**√**	**√**					**√**		**√**	**√**		**√**	**√**	**√**
**IVS II-1, (G>A)**	**√**	**√**	**√**	**√**	**√**	**√**		**√**	**√**	**√**	**√**	**√**	**√**	**√**	**√**		**√**
**CD6, (–A)**	**√**	**√**	**√**	**√**	**√**	**√**							**√**			**√**	**√**
**-101, (C>T)**	**√**	**√**	**√**										**√**				**√**
**-87, (C>G)**	**√**			**√**	**√**							**√**		**√**	**√**	**√**	**√**
**CD5, (–CT)**	**√**			**√**	**√**		**√**	**√**				**√**	**√**	**√**	**√**		**√**
**CD8, (–AA)**	**√**	**√**	**√**	**√**	**√**			**√**	**√**	**√**	**√**		**√**	**√**		**√**	**√**
**IVS I-2, (T>C)**	**√**	**√**	**√**														
**IVS I-5, (G>A)**	**√**		**√**	**√**									**√**				**√**
**IVS I-5, (G>C)**			**√**	**√**		**√**	**√**	**√**	**√**	**√**	**√**	**√**					**√**
**IVS II-848, (C>A)**	**√**		**√**	**√**	**√**						**√**	**√**				**√**	
**-30, (T>A)**	**√**		**√**	**√**	**√**									**√**	**√**		**√**
**Mut. Det. Rate (%)**	97.0	76.8	88.1	84.3	81.2	80.2	64.6	75.8	96.4	48.9	79.6	76.1	73.7	22.9	74.0	97.0	82.4

BG	MK	AL	YU	HR	RO	HU	CZ	DE	DK	NL	GB	FR	ES	PT	IT	RU
18	19	20	21	22	23	24	25	26	27	28	29	30	31	32	33	34
**√**	**√**	**√**	**√**	**√**	**√**		**√**	**√**	**√**	**√**	**√**	**√**	**√**	**√**	**√**	**√**
**√**	**√**	**√**	**√**	**√**	**√**	**√**	**√**	**√**	**√**	**√**	**√**	**√**	**√**	**√**	**√**	
	**√**	**√**	**√**		**√**	**√**	**√**	**√**	**√**	**√**	**√**	**√**	**√**	**√**		**√**
	**√**	**√**	**√**	**√**	**√**	**√**	**√**		**√**	**√**	**√**	**√**	**√**	**√**	**√**	**√**
**√**	**√**	**√**	**√**	**√**	**√**	**√**	**√**	**√**		**√**		**√**	**√**		**√**	
**√**	**√**	**√**	**√**	**√**		**√**	**√**		**√**	**√**		**√**				**√**
**√**	**√**				**√**			**√**		**√**			**√**		**√**	
**√**		**√**								**√**	**√**			**√**	**√**	
**√**	**√**		**√**		**√**			**√**		**√**					**√**	
**√**	**√**				**√**				**√**	**√**	**√**					
**√**	**√**			**√**	**√**				**√**	**√**			**√**			**√**
																**√**
																
		**√**						**√**	**√**	**√**					**√**	
	**√**			**√**						**√**			**√**			
	**√**									**√**						
73.2	95.7	84.8	59.8	63.9	97.5	82.7	86.6	76.5	64.0	66.3	57.8	90.5	84.3	90.0	83.3	69.4

## Supporting Information

S1 Fig TH1amplicon. Normalized and shifted melting curves.
(TIF)

S2 Fig TH2A amplicon. Normalized and shifted melting curves.
(TIF)

S3 Fig TH2B amplicon. Normalized and shifted melting curves.
(TIF)

S4 Fig TH3 amplicon. Normalized and shifted melting curves.
(TIF)

S5 Fig TH4 amplicon. Normalized and shifted melting curves.
(TIF)

S6 Fig TH6-7 amplicon. Normalized and shifted melting curves.
(TIF)

S7 Fig TH5 amplicon. Normalized and shifted melting curves.
(TIF)

S8 Fig XPro amplicon. Normalized and shifted melting curves.
(TIF)

S9 Fig TH1-6-7 multiplexed amplicon. Normalized and shifted melting curves.
(TIF)

S10 Fig TH2B-3 multiplexed amplicon. Normalized and shifted melting curves.
(TIF)

S11 Fig Amplicon TH1. Differentiation plot of -101 (C>T) and -87 (C>G) mutations (A).
(TIF)

S12 Fig Amplicon TH1. Differentiation plot of -101 (C>T) and -87 (C>G) mutations (B)
(TIF)

S13 Fig Amplicon TH3. Differentiation plot of IVS I-110 (G>A) and CD39 (C>T) mutations (A).
(TIF)

S14 Fig Amplicon TH3. Differentiation plot of IVS I-110 (G>A) and CD39 (C>T) mutations (B).
(TIF)

S1 File Statistical analysis of values displayed in Table 7. (XLSX)
