# Supplementary figures and images for: Development of a High-Resolution Melting Approach for Scanning Beta Globin Gene Point Mutations in the Greek and Other Mediterranean Populations
Source: PLoS One. 2016 Jun 28;11(6):e0157393. doi: 10.1371/journal.pone.0157393 (PMC4924799; doi:10.1371/journal.pone.0157393)

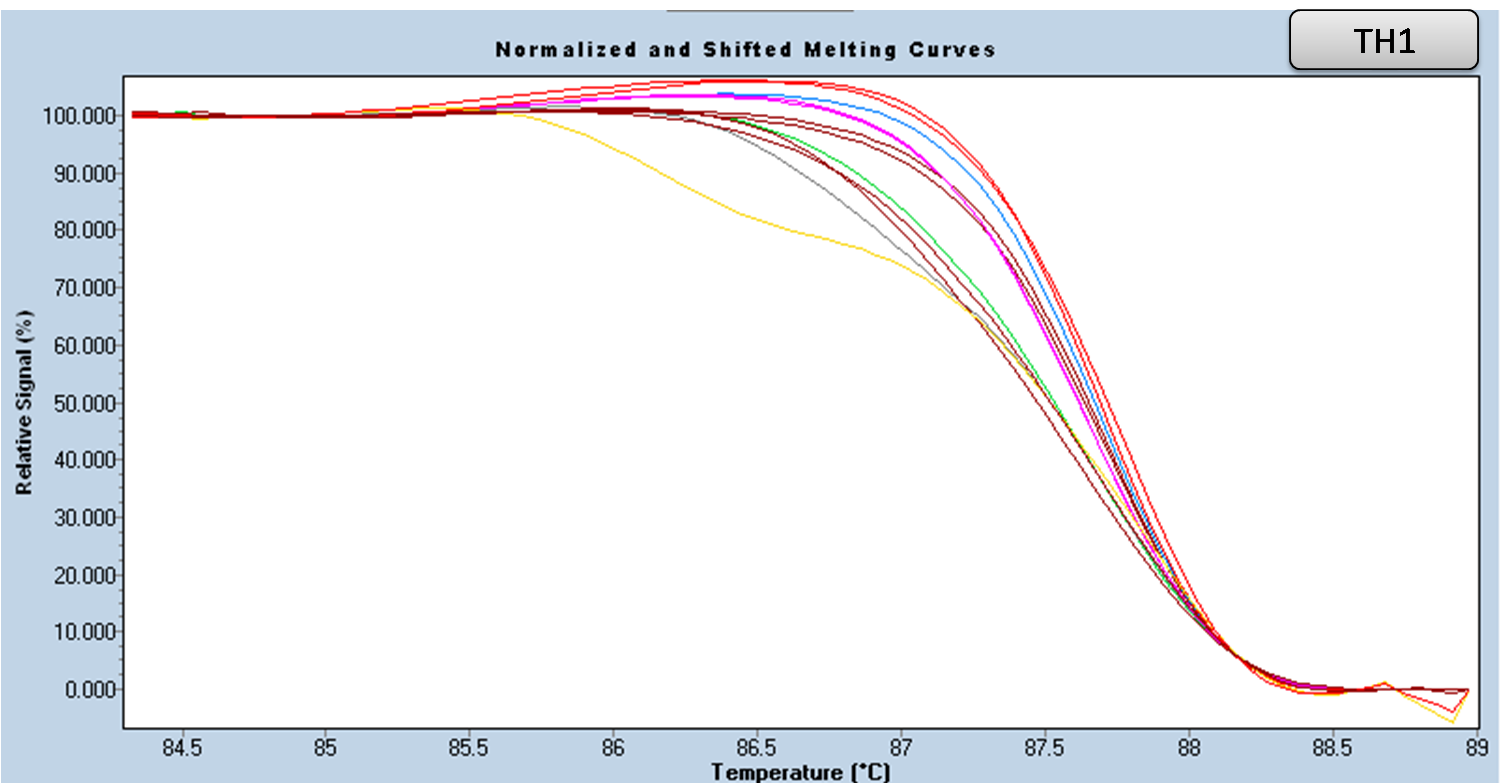

Supplement: S1 Fig — Normalized and shifted melting curves. (TIF) [file pone.0157393.s001.tif]

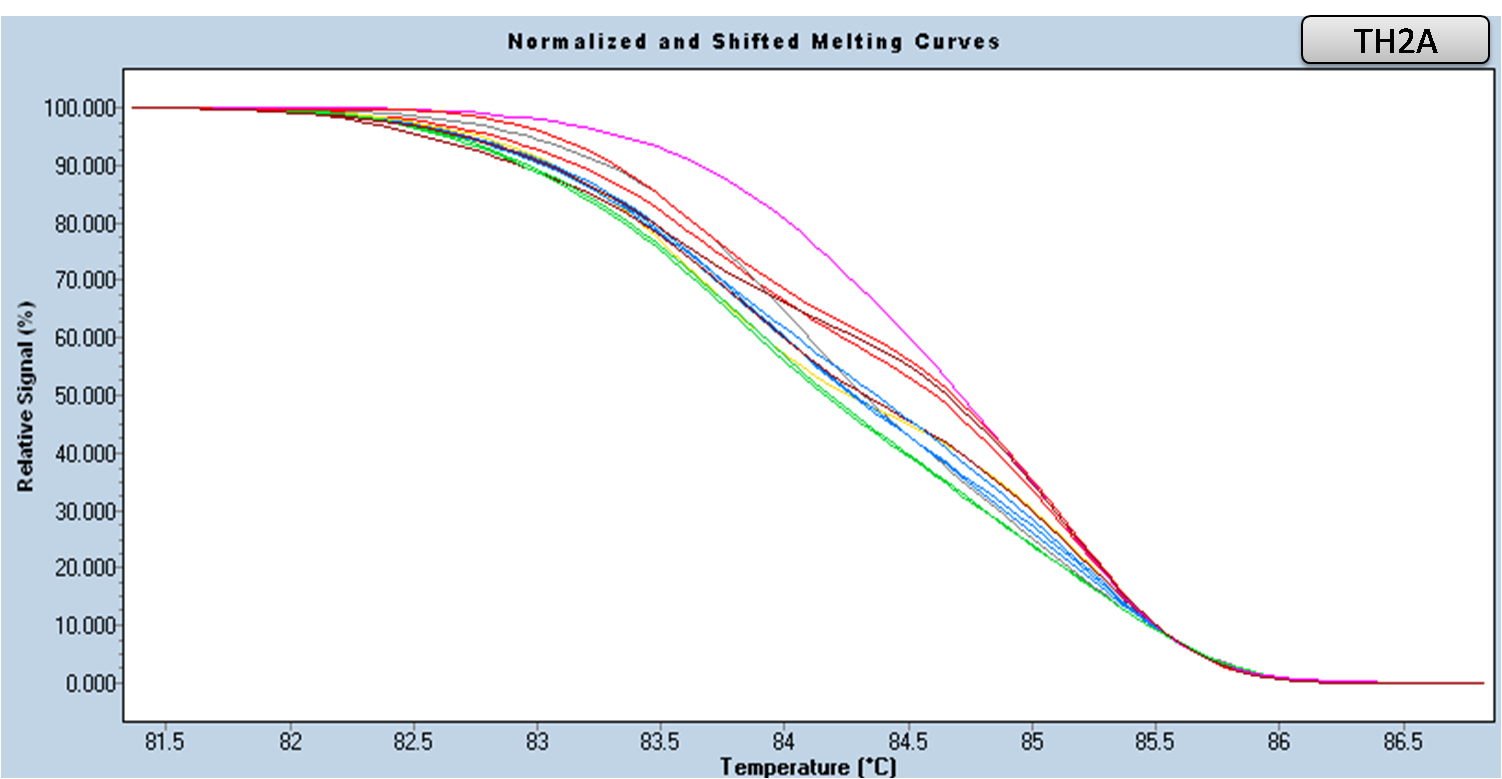

Supplement: S2 Fig — Normalized and shifted melting curves. (TIF) [file pone.0157393.s002.tif]

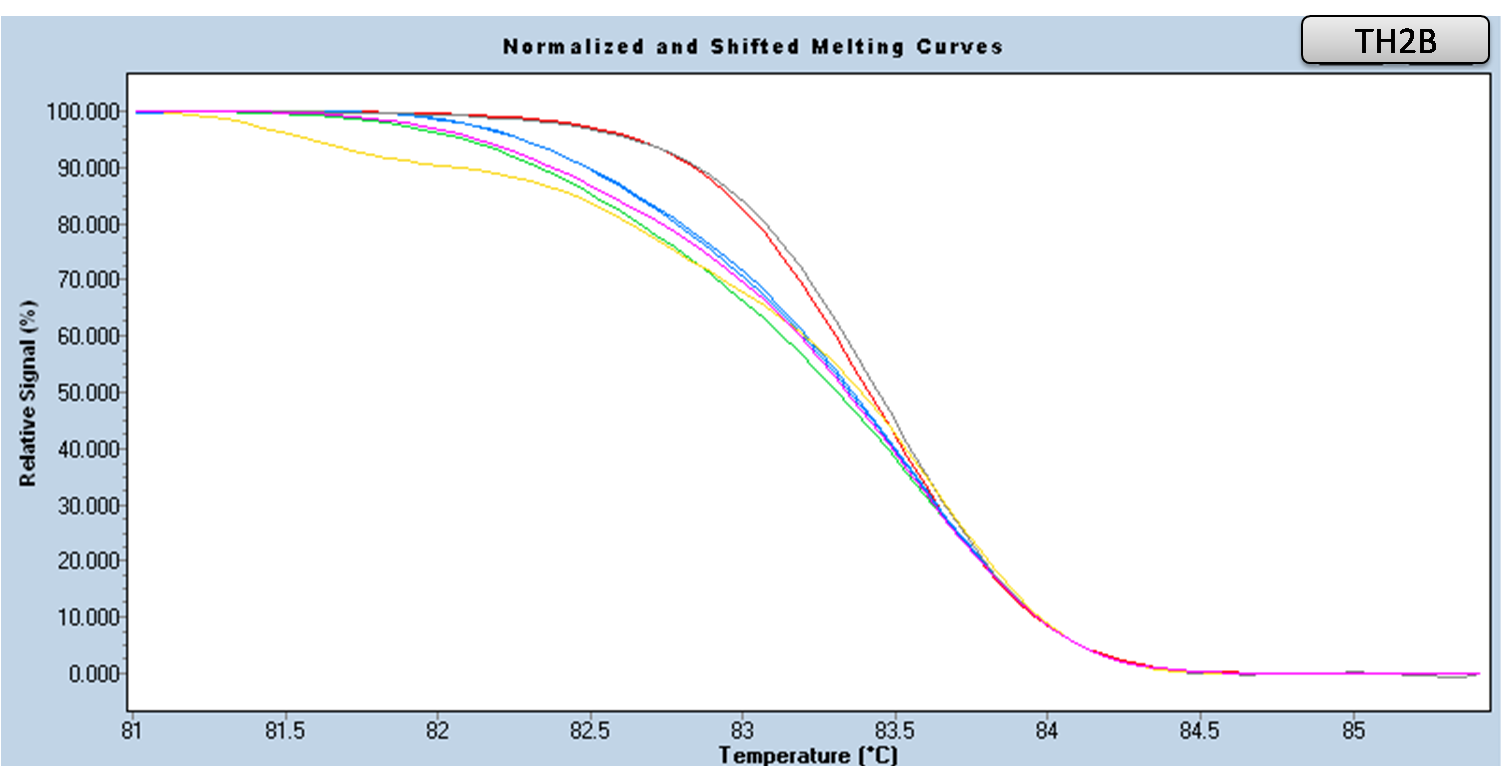

Supplement: S3 Fig — Normalized and shifted melting curves. (TIF) [file pone.0157393.s003.tif]

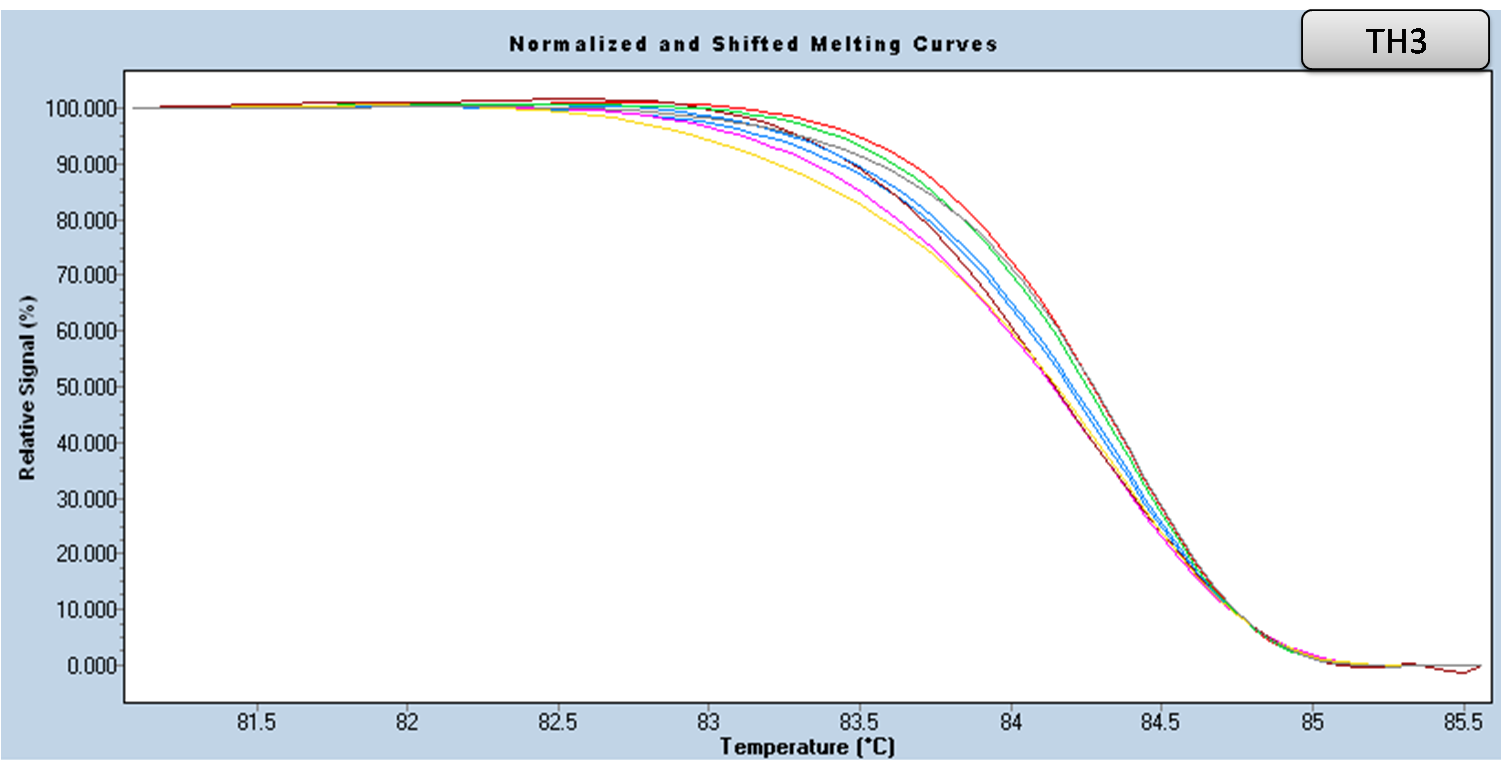

Supplement: S4 Fig — Normalized and shifted melting curves. (TIF) [file pone.0157393.s004.tif]

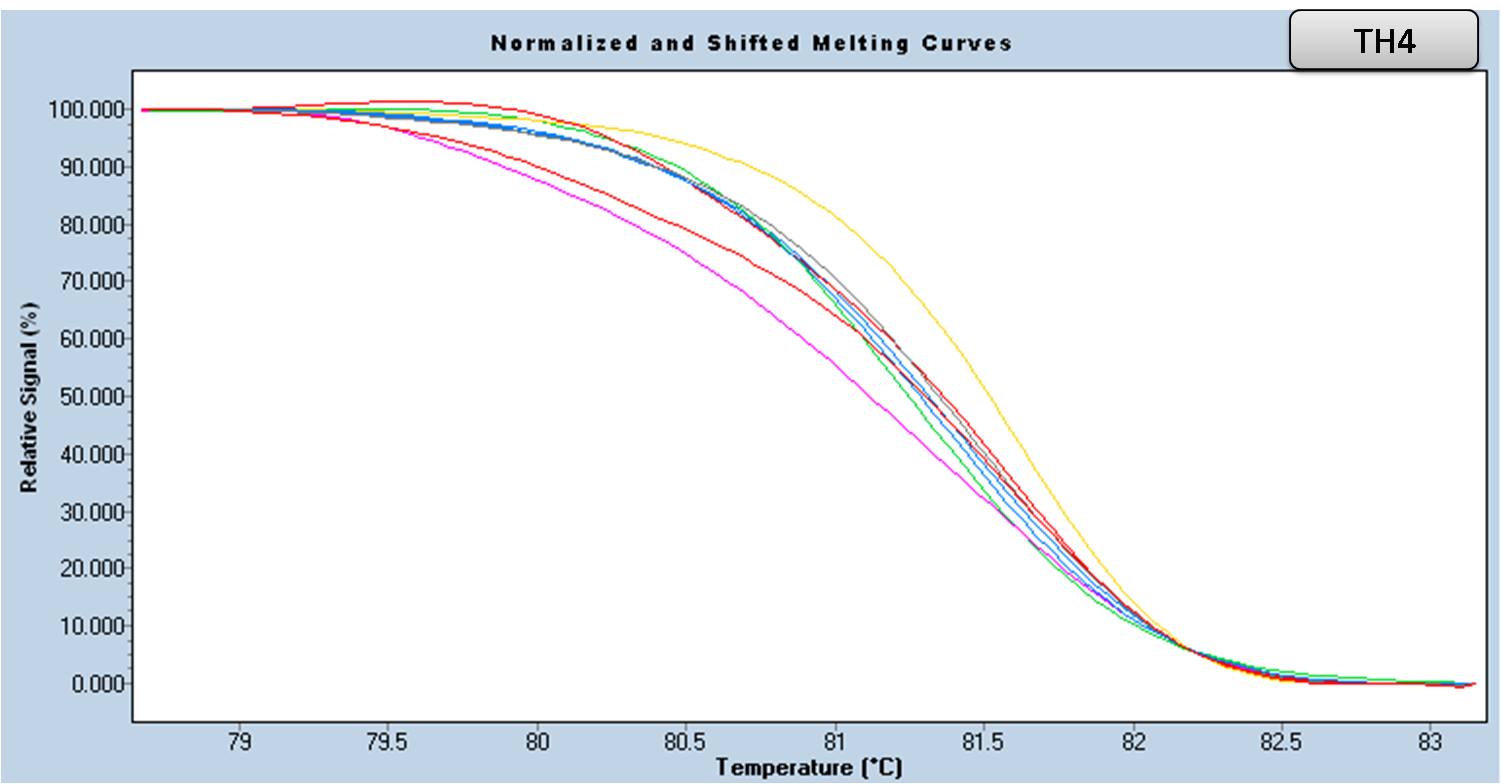

Supplement: S5 Fig — Normalized and shifted melting curves. (TIF) [file pone.0157393.s005.tif]

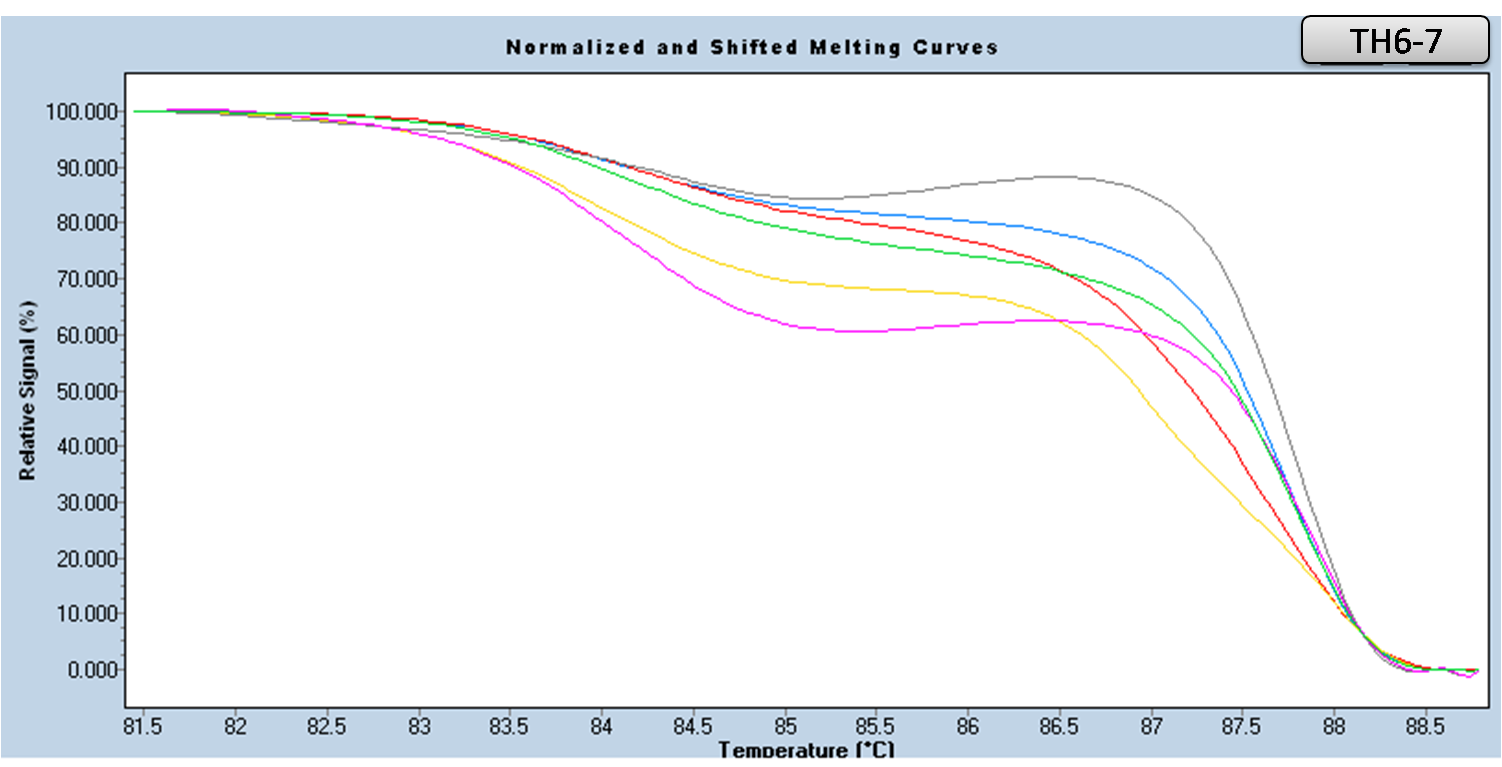

Supplement: S6 Fig — Normalized and shifted melting curves. (TIF) [file pone.0157393.s006.tif]

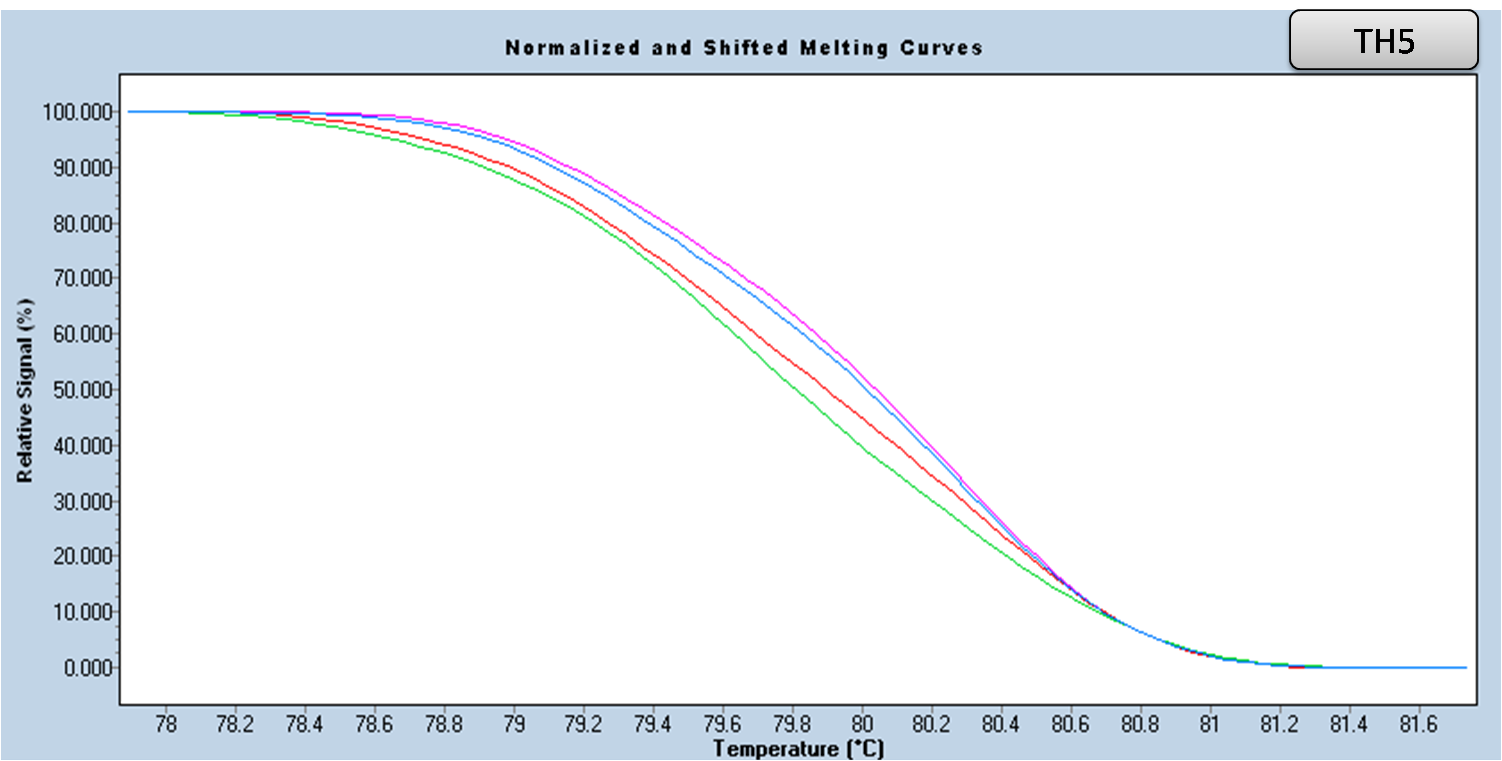

Supplement: S7 Fig — Normalized and shifted melting curves. (TIF) [file pone.0157393.s007.tif]

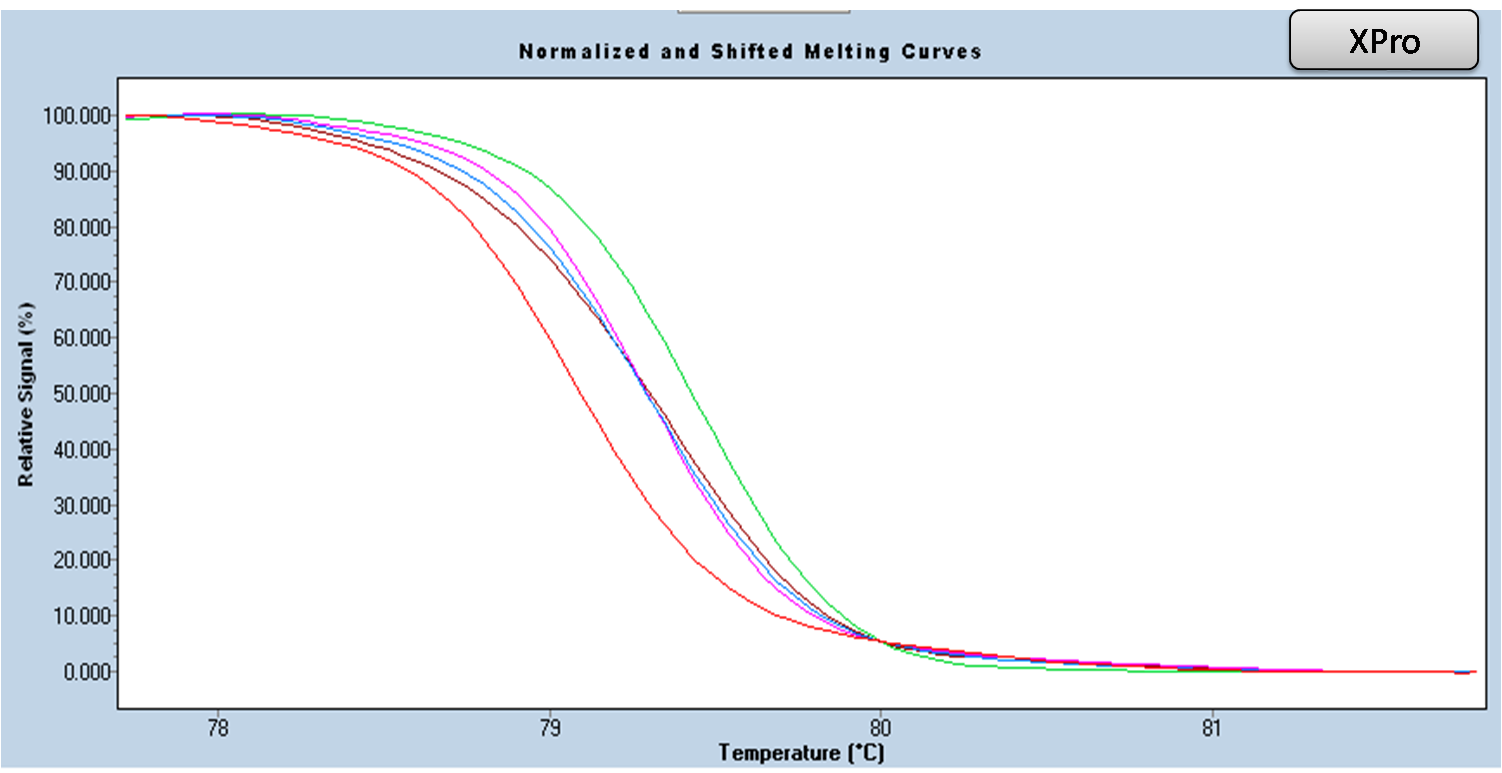

Supplement: S8 Fig — Normalized and shifted melting curves. (TIF) [file pone.0157393.s008.tif]

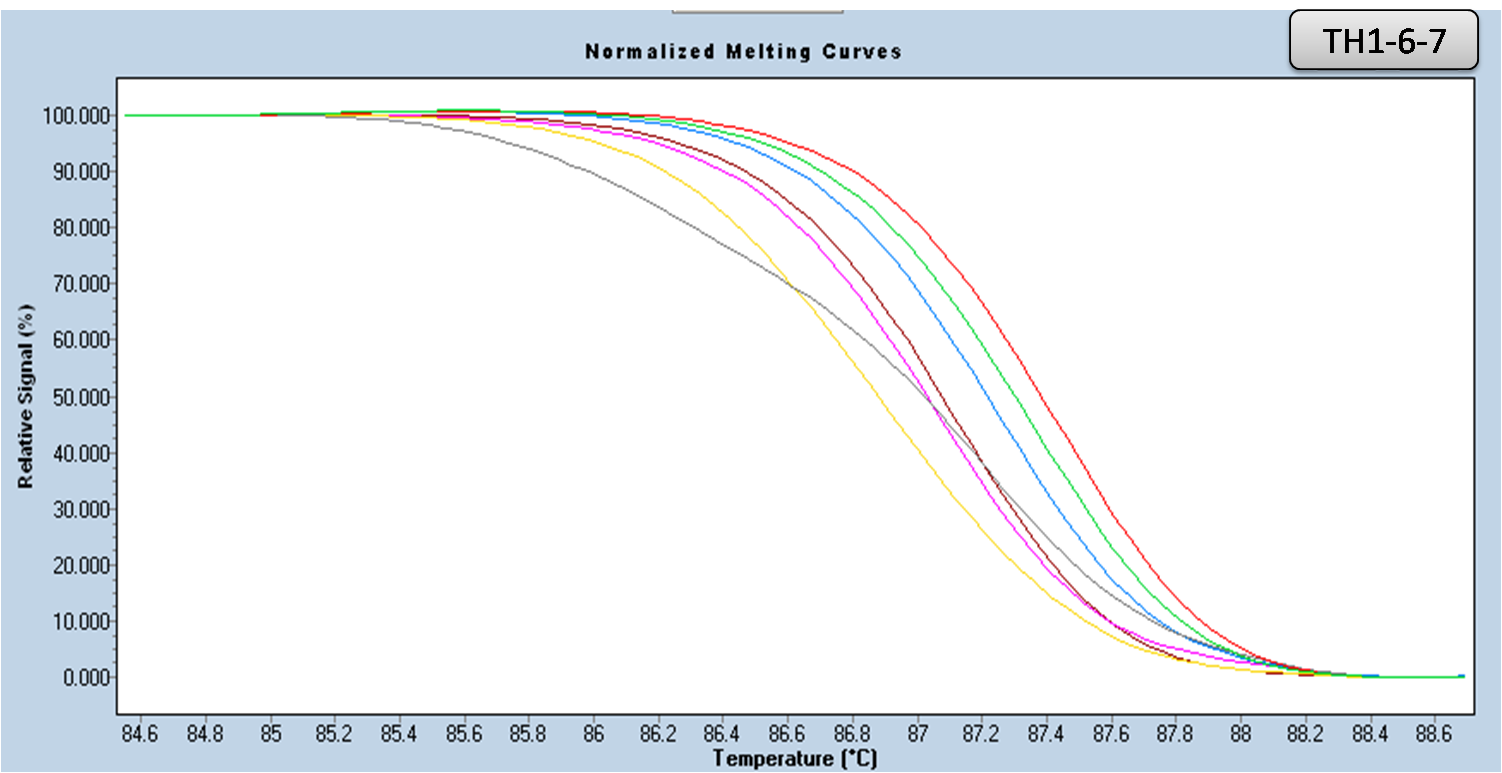

Supplement: S9 Fig — Normalized and shifted melting curves. (TIF) [file pone.0157393.s009.tif]

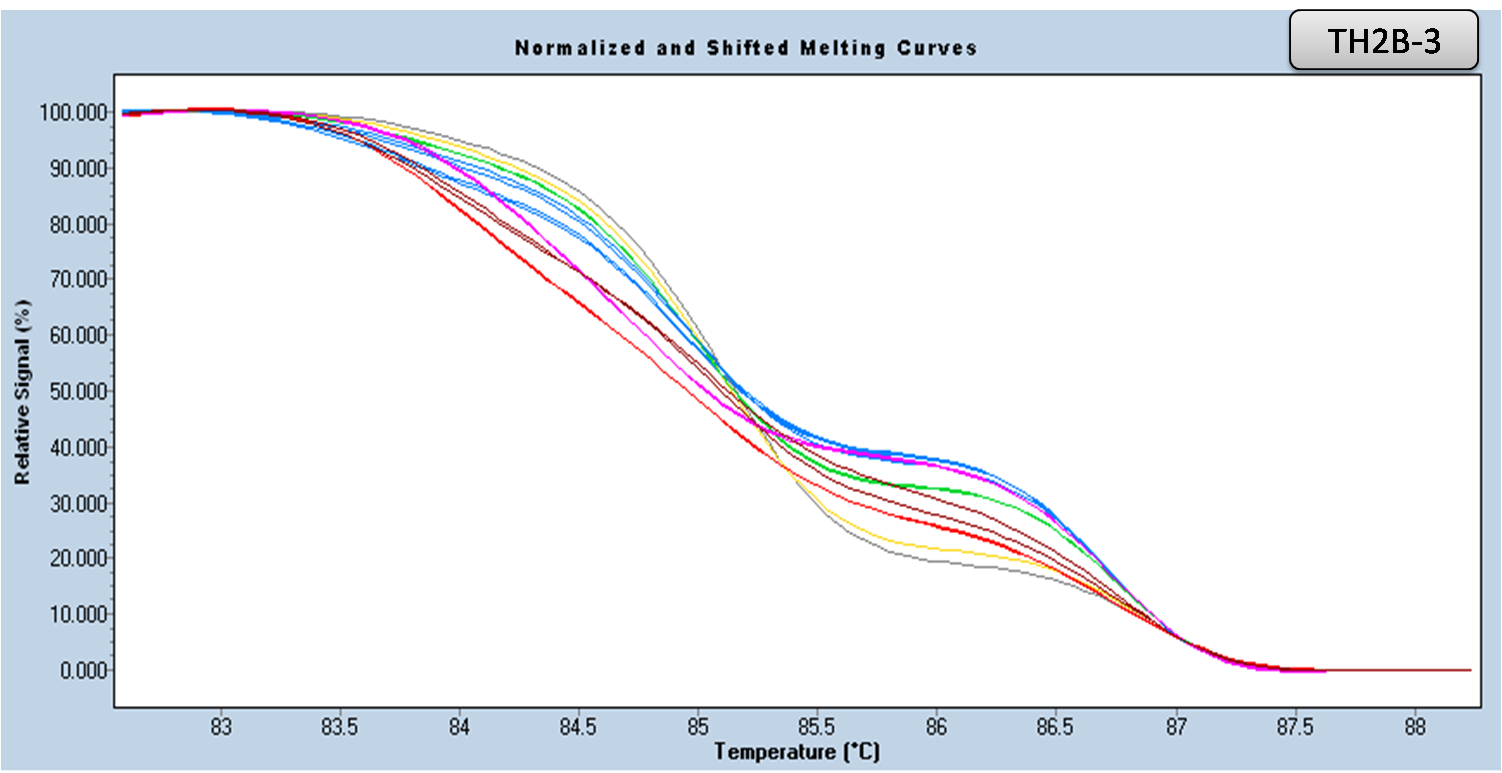

Supplement: S10 Fig — Normalized and shifted melting curves. (TIF) [file pone.0157393.s010.tif]

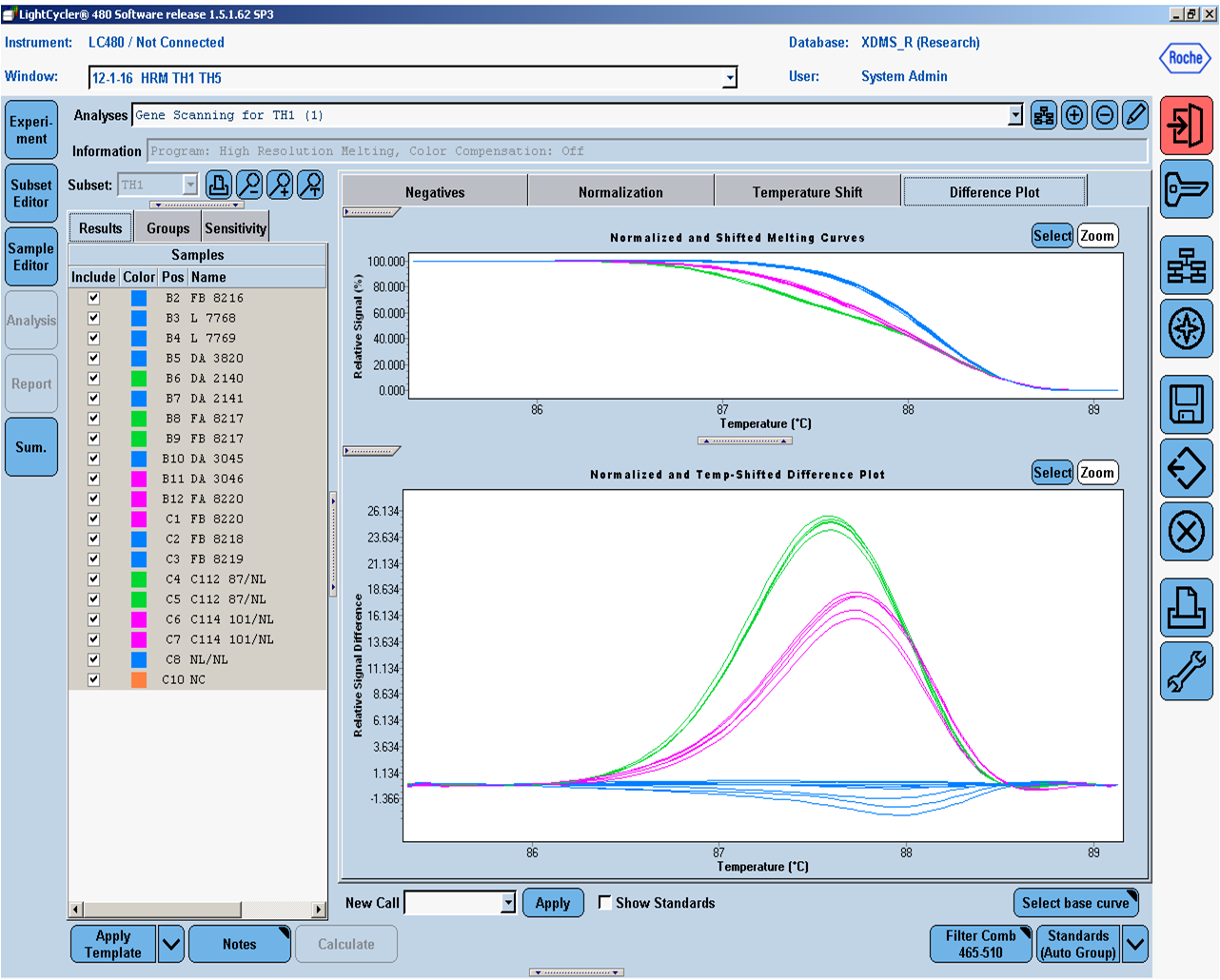

Supplement: S11 Fig — Differentiation plot of -101 (C>T) and -87 (C>G) mutations (A). (TIF) [file pone.0157393.s011.tif]

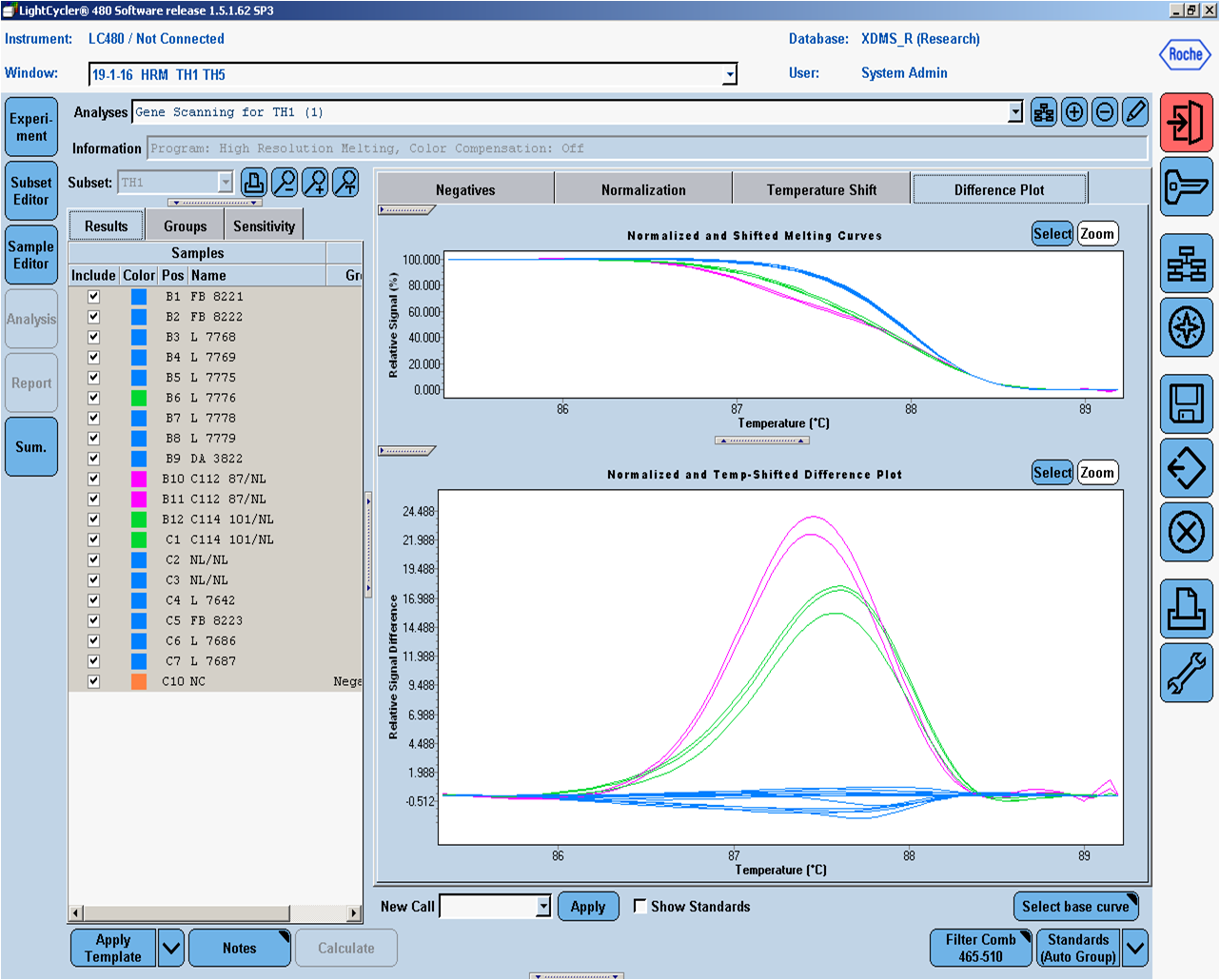

Supplement: S12 Fig — Differentiation plot of -101 (C>T) and -87 (C>G) mutations (B) (TIF) [file pone.0157393.s012.tif]

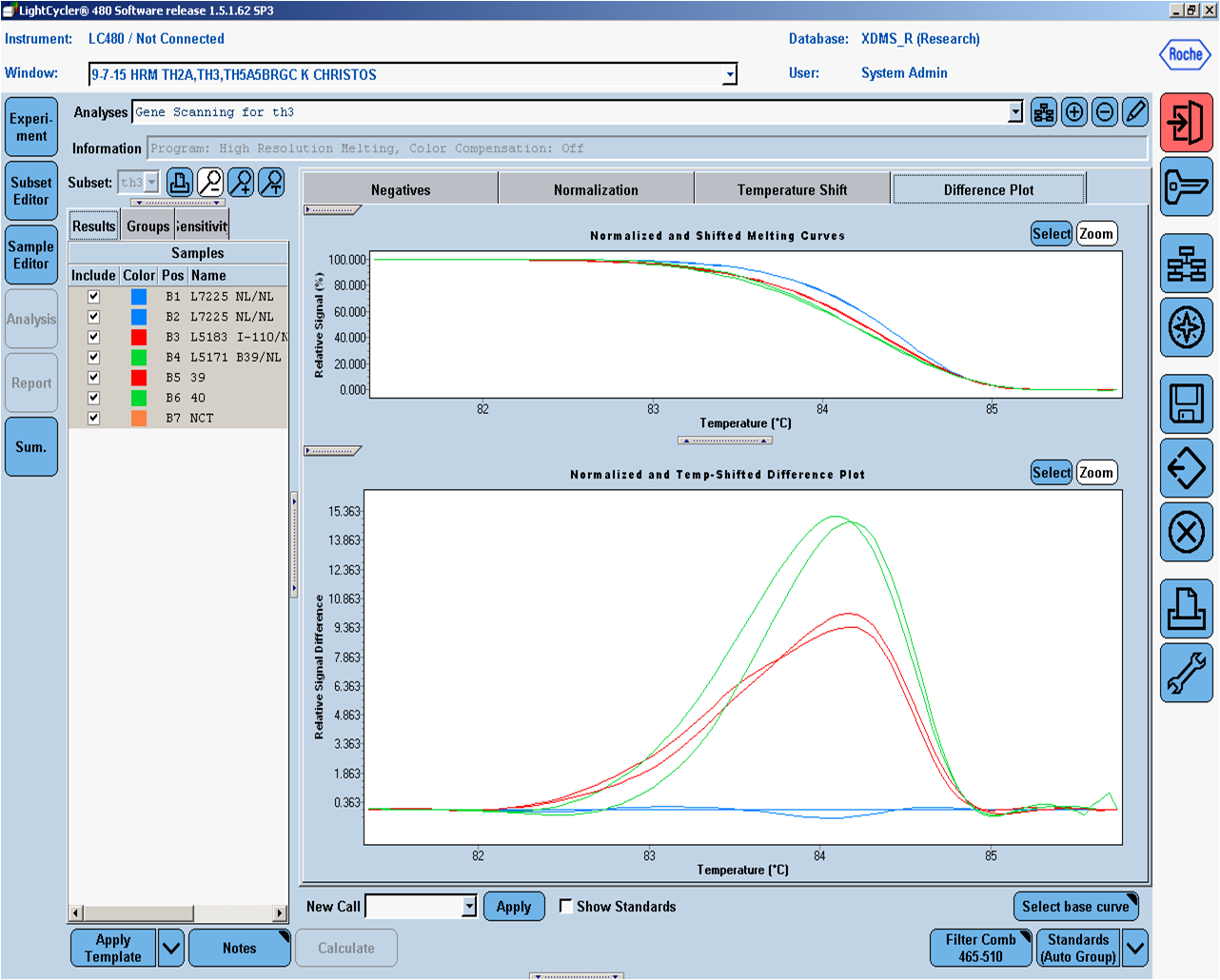

Supplement: S13 Fig — Differentiation plot of IVS I-110 (G>A) and CD39 (C>T) mutations (A). (TIF) [file pone.0157393.s013.tif]

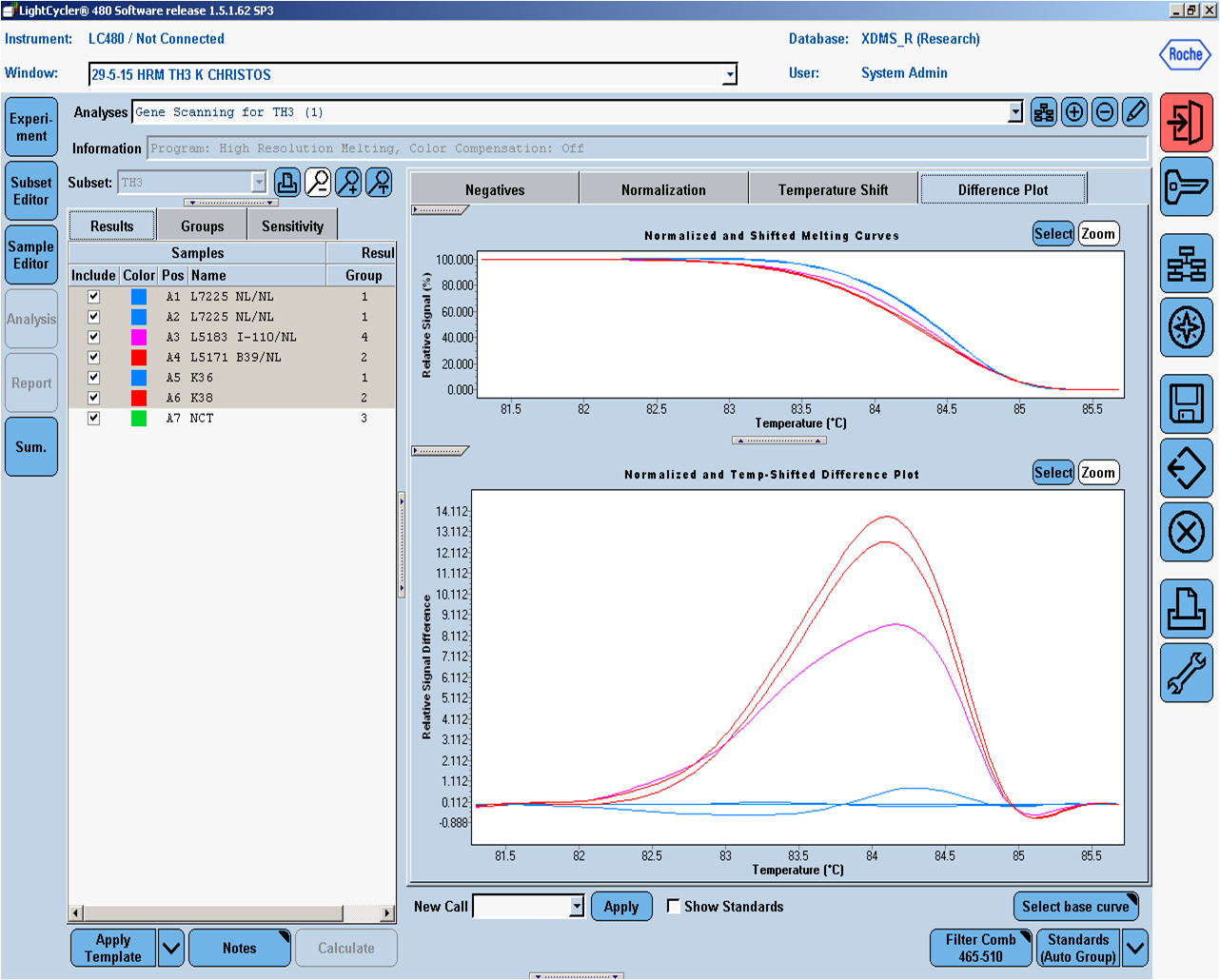

Supplement: S14 Fig — Differentiation plot of IVS I-110 (G>A) and CD39 (C>T) mutations (B). (TIF) [file pone.0157393.s014.tif]
